# Host Factor Induced Bacterial Extracellular Vesicles Promote Horizontal Gene Transfer in *Vibrio cholerae*

**DOI:** 10.1002/jev2.70301

**Published:** 2026-05-09

**Authors:** Dominik Fleischhacker, Yi‐Chi Chen, Noa Sanchez Gordo, Amar Cosic, Ratchara Kalwong, Leo Eberl, Stefan Schild

**Affiliations:** ^1^ Institute of Molecular Biosciences University of Graz Graz Austria; ^2^ Department of Plant and Microbial Biology University of Zurich Zurich Switzerland; ^3^ BioTechMed Graz Graz Austria; ^4^ Field of Excellence Biohealth – University of Graz Graz Austria; ^5^ Austrian Agency for Health and Food Safety (AGES) Institute for Medical Microbiology and Hygiene Graz Austria

**Keywords:** antimicrobial resistance spread, EVs, explosive vesicles, in vivo, OMVs, proteome, vesiduction

## Abstract

Like other Gram‐negative bacteria, *Vibrio cholerae*, releases bacterial extracellular vesicles (BEVs), which have documented roles along the facultative human‐pathogen's lifecycle. Most studies have focused on BEVs released its outermost surface under non‐stressed conditions, which are mainly composed of outer membrane and periplasmic components. Herein, we comprehensively characterise stress‐induced BEVs released upon exposure to the SOS response‐inducing genotoxin mitomycin C or the antimicrobial emulsifier bile, which *V. cholerae* faces during intestinal colonisation. Compared to control BEVs from non‐stressed *V. cholerae* cultures, MMC and bile trigger the release of a high number of enlarged, nucleic acid‐rich BEVs with increased cytoplasmic content, a hallmark of cell lysis‐derived BEVs. Despite similarities between stress‐induced BEVs, our results indicate stressor‐specific BEV compositions and divergent SOS response activation, suggesting different biogenesis routes and subtypes of stress‐induced BEVs. Stress‐induced BEVs promote horizontal gene transfer (HGT) of a chromosomal antibiotic resistance cassette during laboratory cultivation and intestinal colonisation. We provide novel insights in BEV‐mediated HGT, which is independent of the PilA‐pilus of the competence machinery, but requires the periplasmic ComEA complex and downstream components. BEV‐mediated HGT is facilitated under intestinal conditions, that is, exposure to bile and proteases, which highlights the potential of genetic exchange via BEVs during host colonisation.

## Graphical abstract

Antimicrobial stressors, that is, bile or mitomycin C, induce enlarged, nucleic acid‐rich bacterial extracellular vesicles in *Vibrio cholerae* that facilitate horizontal gene transfer in vitro and during intestinal colonisation. The findings provide a detailed comparison of control versus stress‐induced vesicles, identify bile as a host factor shaping vesicle properties, and reveal mechanistic links between vesicle‐mediated genetic exchange and bacterial competence.

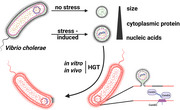

## 1 Introduction

The release of extracellular vesicles is a universal, conserved cellular process that occurs in all domains of life (Gill et al. 2019; Deatherage and Cookson 2012). Since the first reports of bacterial extracellular vesicles (BEVs) emerging from *Escherichia coli* some 60 years ago, the biogenesis, composition and physiological role of these membrane spheres have been the subject of numerous studies (Toyofuku et al. 2019; Toyofuku et al. 2023; Knox et al. 1966). BEVs can support the export of waste products, act as decoys of the bacterial surface to neutralise membrane‐attacking agents and facilitate remodelling of the bacterial surface. Most importantly, BEVs can be packed with various bioactive compounds, including enzymes, toxins, nucleic acids, antimicrobials and signalling molecules, enabling them to participate in nutrient acquisition, host cell modulation as well as intra‐ and interspecies communication (Toyofuku et al. 2019; Toyofuku et al. 2023; Schwechheimer and Kuehn 2015; Kaparakis‐Liaskos and Ferrero 2015). Recent reports indicate that BEVs can spread systemically throughout the body of a potential host (Han et al. 2019; Wispelwey et al. 1989; Modasia et al. 2023). This underscores a pathophysiological relevance of pathogen‐derived BEVs, particularly because they protect their cargo from degradation by external enzymes such as proteases and nucleases and can be effectively internalised by various host cells (Zingl et al. 2021; Dorward et al. 1989; Bielaszewska et al. 2017; Bruno et al. 2020).

BEVs generally reflect the surface composition of the parental cell. Current models suggest two principal routes of BEV formation, that is, blebbing of the outer membrane or via explosive cell lysis followed by self‐assembly of shattered membrane fragments into spherical structures (Toyofuku et al. 2019). In case of Gram‐negative bacteria blebbing‐type BEVs originate from bulging of the outer membrane, ultimately leading to the release of vesicles that enclose periplasmic content. Consequently, such vesicles are enriched with many components of the outer membrane and periplasm and often referred to as outer membrane vesicles (Toyofuku et al. 2023; Zingl et al. 2020). In contrast to blebbing‐type BEVs, which are released by living bacterial cells, explosive‐type BEVs are released during the death of a subset of the bacterial cells, triggered, for example, by genotoxic stress leading to SOS response activation and prophage induction (Toyofuku et al. 2019; Toyofuku et al. 2023; Turnbull et al. 2016; Jiang et al. 1991; Mandal et al. 2021). A well‐established genotoxic agent used to induce explosive‐type BEVs is mitomycin C (MMC), although gentamicin, chloramphenicol (Cm), ciprofloxacin (CIP) and fosfomycin are also well‐documented stressors that activate the SOS response and enhance vesiculation levels in bacteria (Toyofuku et al. 2023; Turnbull et al. 2016; Fulsundar et al. 2014; Li et al. 2024; Devos et al. 2017; Bauwens et al. 2017; Ghosh and Orman 2024). Notably, explosive‐type BEVs may explain the frequent detection of cytoplasmic proteins and nucleic acids in Gram‐negative vesicles (Kadurugamuwa and Beveridge 1996; Dorward and Garon 1990; Bitto et al. 2017; Sjostrom et al. 2015).

The detection of DNA in BEVs reveals another possible route of genetic exchange between bacterial cells, in addition to the extensively studied mechanisms of bacterial conjugation, viral transduction and the uptake of free DNA via transformation (Tran and Boedicker 2017). BEV‐mediated horizontal gene transfer (HGT), also known as ‘vesiduction’, does not require living cells as donors, phage‐specific genes or the internalisation of extracellular, unprotected DNA, but offers protection against nucleases and thermodegradation (Dorward et al. 1989; Soler and Forterre 2020). Vesiduction in Gram‐negative bacteria has been mainly observed for plasmids and phage DNA even across species, while reports for chromosomal DNA transfer remain scarce (Bitto et al. 2017; Tran and Boedicker 2017; Soler and Forterre 2020; Soler et al. 2015; Domingues and Nielsen 2017; Xu et al. 2024; Dell'Annunziata et al. 2021). Nonetheless, it may represent a general mechanism for the exchange of non‐specialised genetic cargo between bacterial species (Tran and Boedicker 2017). Despite recent studies that have advanced our mechanistic understanding and identified requirements for vesiduction, the exact process remains elusive (Fulsundar et al. 2014; Blesa and Berenguer 2015). Although fusion and release of BEV luminal contents into the recipient cell appears to be the primary uptake route, DNA fragments bound to the surface of the BEVs may also play a significant role (Ho et al. 2015).

Among Gram‐negative bacteria, several studies have characterised BEVs derived from the facultative human pathogen *Vibrio cholerae* in terms of BEV biogenesis, composition and pathophysiological roles (Zingl et al. 2021; Zingl et al. 2020; Roier et al. 2016; Altindis et al. 2014; Ebenberger et al. 2024; Langlete et al. 2019; Rasti and Brown 2019; Baryalai et al. 2025; Potapova et al. 2024; Bitar et al. 2019; Reyes‐Robles et al. 2018). *V. cholerae's* lifestyle is characterised by the transition between aquatic reservoirs and the human gastrointestinal tract, being the causative agent of the secretory diarrheal disease cholera with an annual global burden of 3–5 million cases and 100,000 deaths (Clemens et al. 2017; Sack et al. 2004; Nelson et al. 2009; Schild et al. 2008). Between outbreaks persistence in the aquatic environment is facilitated by biofilm formation on chitinous surfaces mainly provided by zooplankton (Silva and Benitez 2016; Schulze et al. 2021). *V. cholerae* can not only efficiently utilise chitin as carbon and nitrogen source but also induces natural competence during growth on chitin, enabling the acquisition of new genetic material via natural transformation (Meibom et al. 2005; Meibom et al. 2004; van Kessel and Camilli 2024).

*V. cholerae* BEVs are an abundant component the extracellular biofilm matrix and contribute to cell‐to‐cell interactions as well as interbacterial signalling that promotes the formation of a vital biofilm (Ebenberger et al. 2024; Potapova et al. 2024). Upon oral ingestion with contaminated food or water *V. cholerae* induces the virulence cascade via activation of the ToxR regulon by environmental stimuli (Childers and Klose 2007). This leads to the expression of the two main virulence factors, tha tis the cholera toxin and the toxin‐coregulated pilus, both of which are essential for the diarrheal symptoms and successful colonisation of the small intestine (Holmgren and Svennerholm 1977; Taylor et al. 1987). Medium‐induced activation of the virulence cascade under microaerophilic conditions in AKI broth provides a unique experimental advantage, as it allows to study virulence inducing conditions during in vitro cultivation (Iwanaga and Yamamoto 1985; Iwanaga et al. 1986). In order to achieve colonisation fitness, *V. cholerae* also remodels its surface to adapt to in vivo stressors, such as antimicrobial peptides or bile (Provenzano and Klose 2000; Hankins et al. 2012). Enhanced release of BEVs upon host entry facilitates the remodelling and accelerates adaptation to the host environment (Zingl et al. 2020). During intestinal colonisation, BEVs can deliver active cholera toxin to host cells via porin‐dependent uptake (Zingl et al. 2021). Thus, BEVs fulfil multiple physiological functions throughout the lifecycle of *V. cholerae*. Notably, however, most studies to date have focused on blebbing‐type BEVs, while stress‐induced BEVs derived from *V. cholerae* remain largely understudied. However, bacteria constantly face stressors during environmental or in vivo lifestyles, such as nutrient limitations, temperature shifts, UV light or antimicrobial agents. Thus, stress‐induced BEVs may be a common type of vesicle released by Gram‐negative bacteria, particularly facultative pathogens, which encounter various stressors throughout the diverse stages of the lifecycle.

In this study, we investigated BEVs released by *V. cholerae* upon exposure to two stressors: the genotoxic agent MMC, which triggers explosive‐type BEV formation in various bacteria (Turnbull et al. 2016; Toyofuku et al. 2017; Andreoni et al. 2019), two antibiotics with different modes of action, that is, Cm and CIP and the host‐derived emulsifier bile, an antimicrobial stressor encountered during intestinal colonisation. Stress‐induced and control BEVs were isolated from *V. cholerae* cultures grown under virulence inducing conditions (AKI) and non‐virulence inducing conditions (LB) to enable comprehensive comparative analyses. Compared to control BEVs, the stress‐induced BEVs showed increased levels of cytoplasmic proteins and nucleic acids. The latter facilitated BEV‐mediated HGT of a chromosomally encoded antibiotic resistance gene in vitro as well as during host colonisation. Our findings provide new insights into microbial evolution and spread of antibiotic resistance genes, highlighting the role of stress‐induced BEVs in driving genetic exchange.

## 2 Materials and Methods

### 2.1 Bacterial Strains and Growth Conditions

Bacterial strains and plasmids used in this study are listed in Table S1. Oligonucleotides are listed in Table S2. All experiments used the clinical isolate *V. cholerae* O1 El Tor AC53 as the WT strain. Unless stated otherwise, *V. cholerae* strains were cultivated in lysogeny broth (LB) or on LB agar at 37°C. In addition, AKI broth at 37°C was used to induce the ToxT‐dependent virulence cascade (Iwanaga and Yamamoto 1985) and a mixture of ‘Super Optimal broth with a Catabolite repression’ (SOC): HEPES (1:1) was used for HGT assays (Sun et al. 2009). *E. coli* strains DH5αλpir and SM10λpir were used for genetic manipulations and grown with aeration in LB broth or on LB agar at 37°C (Miller and Mekalanos 1988; Miller 1972). Antibiotics and supplements were used at the following final concentrations: streptomycin (Sm), 100 µg mL^−1^; ampicillin (Ap), 100 µg mL^−1^ or 50 µg mL^−1^ in combination with other antibiotics; tetracycline (Tet), 0.5 µg mL^−1^; Cm, 2.0 µg mL^−1^; sucrose, 10%.

### 2.2 Constructions of Mutant Strains

The construction of suicide plasmids and the subsequent generation of deletion mutants were carried out as described previously (Pressler et al. 2016; Seper et al. 2011). Qiagen plasmid kits were used for the isolation of plasmid DNA, Qiaquick Gel extraction and Qiaquick PCR Purification kits (Qiagen) were used for purifying DNA fragments. PCR reactions for subcloning were carried out using the Q5 High‐Fidelity DNA Polymerase (NEB), while Taq DNA Polymerase (New England Biolabs) was used for all other PCRs.

In‐frame deletion mutants were constructed following the method described by Donnenberg and Kaper (1991). Briefly, ∼800 bp PCR fragments located up‐ and down‐stream of the respective gene were amplified using the oligonucleotide pairs X_Y_1 and X_Y_2 as well as X_Y_3 and X_Y_4 (Table S2), where X represents the gene and Y the respective restriction site. After digestion of the PCR fragments with the appropriate restriction enzymes (New England Biolabs) indicated by the name of the oligonucleotide, they were ligated into pCVD442, which was digested with the appropriate restriction enzymes. In the case of pVC1620/1::*tetR* for insertion of a TetR resistance cassette in the intergenic region between the open reading frames VC1620 and VC1621, the *tetR* gene was amplified by the oligonucleotide pair TetR_KpnI and TetR_EcoRI using pBR322 as template. In parallel, flanking ∼800 bp gene fragments located up‐ and down‐stream of the insertion site were amplified using the oligonucleotide pairs VC1620/1_SacI_1 and VC1620/1_KpnI_2 as well as VC1620/1_EcoRI_3 and VC1620/1_XbaI_4. In the case of pVC1620/1::*cmR* for insertion of a CmR resistance cassette in the intergenic region between the open reading frames VC1620 and VC1621, the *cmR* gene was amplified by the oligonucleotide pair CmR_KpnI and CmR_BamHI using pBAD33 as template. The ∼800 bp flanking gene fragments were amplified using the oligonucleotide pairs VC1620/1_SacI_1 and VC1620/1_KpnI_2 as well as VC1620/1_BamHI_3 and VC1620/1_XbaI_4. After digestion of the PCR fragments with the appropriate restriction enzymes indicated by the name of the oligonucleotide, they were ligated into a SacI/XbaI opened pCVD442.

Unless noted otherwise, ligation products were transformed into *E. coli* DH5αλpir and Tet^R^/Ap^R^ colonies (in case of pVC1620/1::*tetR*) or Cm^R^/Ap^R^ colonies (in case of pVC1620/1::*cmR*) or Ap^R^ colonies (in all other cases) were analysed for the correct constructs by PCR. To obtain deletion strains generated derivatives of pCVD442 were transformed into *E. coli* Sm10λpir and conjugated into *V. cholerae*. Exconjugants were purified by Sm^R^/Ap^R^ selection. Sucrose selection was used to obtain Ap^S^ colonies and chromosomal deletions/replacements were confirmed by colony PCR. VC1620/1::*tetR* was further tested for growth on LB plates with Tet (LB‐Tet); VC1620/1::*cmR* was further tested for growth on LB plates with Cm (LB‐Cm).

### 2.3 Isolation of Bacterial Extracellular Vesicles (BEVs)

BEVs were generally isolated as described previously with some modifications (Schild et al. 2009). Overnight (O/N) cultures of the respective strains were grown in LB and diluted to a starting optical density at a wavelength of 600 nm (OD_600_) of 0.02 either in AKI broth (virulence inducing condition) or LB (non‐virulence inducing conditions). AKI and LB cultures were initially grown for 8 h anaerobically in fully filled 500 mL glass bottles, subsequently transferred in 2 L culture flasks and grown for 8 h at 37°C and 120 rpm with aeration in presence or absence of stressors [MMC (Sigma–Aldrich), bile salts (Sigma–Adrich), Cm (Roth) and CIP (AppliChem)] using the indicated concentrations. Bacterial cells were then removed from the supernatant by centrifugation (9,000 × *g*, 15 min) and subsequent sterile filtration (0.22 µm). Removal of living cells was routinely confirmed by plating 1 mL of filter‐sterilised supernatant on LB plates, which did not yield in colony formation after O/N incubation. BEVs present in the filter‐sterilised supernatant were pelleted through subsequent ultracentrifugation (150,000 × *g*, 4°C, 4 h) and resuspended in appropriate volumes of HEPES buffer (10 mM, pH 7.4) to generate a BEV suspension 1000‐fold more concentrated than in the original filter‐sterilised supernatant. Aliquots of BEVs were stored at −20°C for qualitative and quantitative assays or at −80°C for OMIC‐based analyses as well as for HGT experiments. All BEVs used in this study were routinely characterised by a variety of qualitative and quantitative assays, that is, visualisation by TEM, total protein biomass, mean and mode particle size, particle amount, LPS content, lipid content and nucleic acid content.

### 2.4 Density Gradient Purification of BEVs

Density gradient purifications were performed according to Roier et al. (2015). Briefly, BEVs (approximately 1,000 µg protein equivalent) were applied onto an isopycnic OptiPrep‐iodixanol (Sigma–Aldrich) gradient (1.4 mL each of 55%, 50%, 45%, 40%, 35%, 30% and 25% iodixanol in ddH_2_O) and subjected them to ultracentrifugation (150,000 × *g*, 17 h, 4°C, SW 41 Ti rotor). Gradient fractions were collected from top (Fraction 1) to bottom (Fraction 9) under flashlight illumination. Fraction 1 comprised approximately 1 mL up to the visible opaque band, Fraction 2 included ∼0.3 mL encompassing the visible opaque band by flashlight illumination, Fractions 3–8 comprised ∼1.3 mL each and Fraction 9 contained the remaining ∼1.1 mL. Each gradient fraction was diluted to 35 mL in HEPES buffer (10 mM, pH 7.4), pelleted by ultracentrifugation (144,000 × *g*, 4 h, SW 32 Ti rotor) and resuspended in HEPES buffer using 120 µL for visible pellets and 50 µL for non‐visible pellets. Purified fractions were subjected to protein and nucleic acid quantification using Bradford and SYTO 9 assays. Fraction 2 containing detectable amounts was furthermore analysed for nanoparticle size and amounts.

### 2.5 Protein, Nucleic Acid, Lipid and Lipopolysaccharide (LPS) Quantification

Amounts of diverse biomolecules in BEV samples were determined as previously described (Zingl et al. 2020; Roier et al. 2016; Ebenberger et al. 2024; Thapa et al. 2023). In detail, protein concentrations were determined by Bradford assays (Bio‐Rad Laboratories Inc., Protein Assay Dye Reagent) according to the manufacturer's manual using BSA (Roth) dissolved in HEPES buffer (10 mM, pH 7.4) as standard. To ensure detection of luminal content of vesicles, samples were lysed with 0.1% SDS for 10 min before the assay. Nucleic acid concentrations were determined by using the cell membrane permeable dye SYTO 9 Green‐Fluorescent Nucleic Acid Stain (Invitrogen) according to the manufacturer's manual as previously described. As standard chromosomal DNA isolated from *V. cholerae* WT was used, which was dissolved in HEPES buffer (10 mM, pH 7.4) and quantified by Nanodrop 100 (Thermo Scientific). Lipid quantification was performed using FM 4–64 (*N*‐(3‐triethylammoniumpropyl)−4‐(6‐(4‐(diethylamino) phenyl) hexatrienyl) pyridinium dibromide). The LPS content of BEVs was determined via purpald assays using 3‐deoxy‐d‐mannooctulosonic acid (Sigma–Aldrich) as a standard.

### 2.6 Size and Nanoparticle Amount Measurement

Size distributions of the isolated BEVs were assessed by dynamic light scattering (DLS) using the Zetasizer Nano ZS90 (Malvern, UK) as previously described with minor modifications (Zingl et al. 2021; Ebenberger et al. 2024). Samples were diluted 1:1000 in filtered HEPES buffer (10 mM, pH 7.4) using an alumina‐based membrane with a pore size of 0.02 µm (Anotop) and processed at 25°C under standard settings [dispersant refractive index = 1.331, viscosity (cP)  =  0.89]. The average BEV size of each sample was determined by three independent measurements, with 21–28 cycles for each measurement, using a measurement angle of 173° (backscatter), auto measurement duration and ‘seek for optimal position’ as positioning setting. Nanoparticle concentrations in BEV samples were determined by a NanoSight NS300 instrument (Malvern Panalytical Ltd, UK) with NTA 3.4 software. BEVs were diluted in aqua bidest (Fresenius Kabi) to a final concentration of 50–150 particles per frame before being measured in the light scattering mode with a laser 488 module. For each sample, ten 60‐s videos were recorded in the camera level 16.

### 2.7 Protein Profile Analysis via SDS‐PAGE and Silver Staining

Qualitative differences in protein composition of BEVs were visualised by sodium dodecyl sulphate‐polyacrylamide gel electrophoresis (SDS‐PAGE) using polyacrylamide (12%) gels in combination with the Mini‐PROTEAN Tetra cell system (Bio‐Rad) (Laemmli 1970). Before approximately 2 µg of BEV sample (protein equivalent determined by Bradford) were loaded on the gels, the BEVs were diluted to 0.1 µg/µL in HEPES buffer (10 mM, pH 7.4), mixed with Laemmli buffer and incubated for 20 min at 95°C to achieve proper denaturation. As molecular mass standard the PageRuler Prestained Protein Ladder 10 to 180 kDa (Thermo Fisher Scientific) was used as indicated. Subsequently protein bands were visualised by silver staining as previously described (Zingl et al. 2021).

### 2.8 Cholera Toxin (CT) ELISA

GM1 ELISA was used to quantify the concentration of CT in cell‐free supernatant samples as described previously (Zingl et al. 2021; Vorkapic et al. 2019).

### 2.9 SOS Stress Response Assays

Induction of a SOS stress response in *V. cholerae* upon exposure to MMC or bile salts was assessed by fluorescence‐based reporter system using the plasmid precN‐gfp, which carries the green fluorescent protein (GFP) gene fused to the *recN* promoter (Baharoglu and Mazel 2011). RecN expression is upregulated during SOS induction and has been previously used to analyse SOS induction in *V. cholerae* by subinhibitory concentrations of diverse antibiotics (Baharoglu and Mazel 2011; Baharoglu et al. 2010). Overnight (O/N) cultures of the respective strains were grown in LB and diluted to a starting OD_600_ of 0.02 either in AKI broth (virulence inducing condition) or LB (non‐virulence inducing conditions). AKI and LB cultures were initially grown for 8 h anaerobically and subsequently transferred in a black 24‐well microtiter plate (Sensoplate, F glass bottom, Greiner Bio‐One) with 1 mL per well in presence or absence of stressors, that is, MMC (Sigma–Aldrich) or bile salts (Sigma–Aldrich). A media only control well served as blank. The 24‐well plate was placed in a microplate reader (FLUOstar Omega, BMG LabTech) at 37°C with aeration achieved by 300 rpm shaking. OD_600_ and fluorescence (excitation: 485 nm/emission: 520 nm) were measured every 30 min for 8 h. Ratio was calculated as follows: fluorescence (RFU)/OD_600_.

### 2.10 Transmission Electron Microscopy

To visualise the BEVs by transmission electron microscopy (TEM) appropriate dilutions in aqua bidest (Fresenius‐Kabi) were allowed to adsorb on a pre‐glow‐discharged (15 mA 25s PELCO easiGlowTM) formvar‐coated 300‐mesh copper grid (Plano GmbH, SF162‐3). After 1 min, the excess liquid was removed using filter paper. The grid was negatively stained with 1% uranyl acetate for 1 min and dried with filter paper. Electron micrographs were recorded using a Tecnai G2 transmission electron microscope (FEI, Eindhoven, Netherlands) with a High Speed CMOS camera Gatan Rio 16. Acceleration voltage was 120 kV.

### 2.11 Qualitative and Quantitative Proteomic Analyses

Protein quantification via mass spectrometry (MS) was conducted at the Functional Genomic Center Zurich (FGCZ) at the University of Zurich (UZH). Sample preparation for label‐free quantitative proteomics was done according to the FASP method as described previously (Wiśniewski et al. 2009). In brief, 20 µg of protein of each MV sample was quantified using a Qubit Protein Assay kit (Thermo Fisher Scientific). After denaturation of with 4% SDS, 0.1 M DTT, 8 M urea, 100 mM Tris, pH 8.2 at 95°C for 5 min the sample was subjected to IAA alkylation. Trypsin (0.4 µg; Promega) was added overnight, and the digested peptides were then acidified by adding trifluoracetic acid to a final concentration of 0.5%. Following desalting with C18 stage tips, the samples were freeze‐dried and stored at −20°C before LC‐MS analysis (Universal sample preparation method for proteome analysis. Nature Methods, 6, 359–362). For LC‐MS data acquisition, iRT peptides (Biognosys) were added to the samples for calibration. Peptides were separated on an ACQUITY UPLC M‐Class System (Waters) equipped with a HSS T3 C18 reverse‐phase column (1.8 µm, 75 µm × 250 mm, Waters) and analysed by an Orbitrap Fusion Lumos Tribrid mass spectrometer (Thermo Fisher Scientific). Data were acquired with the DDA mode using Solvent A (0.1% formic acid in H_2_O) and Solvent B (0.1% formic acid in acetonitrile) with a 108 min gradient; 5% B for 3 min, 5%–22% B in 80 min, 22%–32% B in 10 min, 32%–95% B in 5 min, 95% B for 10 min with a column temperature of 50°C and a constant flow rate of 0.3 µL/min. Thermo raw files were converted to the Mascot generic format (MGF) by the Proteome Discoverer (v2.0; Thermo Fisher Scientific) using the automated rule‐based converter control (Barkow‐Oesterreicher et al. 2013). Mascot search was done against the *V. cholerae* O1 proteome database obtained from UniProt combined with common contaminants and decoys with the following parameters: fixed modification carbamidomethyl (C) and variable modification deamidated (NQ) and oxidation (M); max cleavage 1; peptide charges 2+, 3+, 4+; peptide tolerance 10 ppm; MS/MS tolerance 0.6 Da. The GO Annotations Database was used for protein location prediction. Progenesis QI software (v4.2; Waters) was used for quantitative proteomic analysis. Alignment of chromatograms was carried out by combining automatic and manual alignment with iRT peptide standards. Peptides with MS/MS spectra ranking greater than 6 were excluded. Peptide and protein identification was done by Scaffold 5 (Proteome Software) with Mascot searching and was imported back to Progenesis. All experiments were done in three biological replicates. Proteins absent in 2 of 3 replicates for all three BEV types or with peptide counts < 2 were excluded.

Changes in the amount of proteins between two sample preparations were visualised using Violin plots or are provided as individual ratios for selected SOS‐regulon associated proteins. Therefore, the individual ratios of the mean ‘score’ values of all proteins identified in the two sample preparations were calculated as follows: mean ‘score’ value of the protein in sample Preparation 1 divided by mean ‘score’ value of the protein in sample Preparation 2.

### 2.12 DNA Sequencing and Analyses

To remove non‐luminal DNA in the BEV samples, 110 µL of DNase (10x) buffer was added to 3.2–10.0 × 10^12^ of BEVs and digested with 10 µL recombinant DNase I (RNase‐free, 10 U µL^−1^, Roche) at 37°C for 10 min followed by heat inactivation at 80°C for 25 min. For each condition, DNase‐treated BEV samples derived from 6 to 8 independent biological cultures were pooled before downstream processing to ensure sufficient material (resulting in the sequencing analysis of *n* = 1 per condition). Luminal DNA was isolated via DNeasy Blood & Tissue Kit (QIAGEN 69504). To ensure the quality and quantity of the sample for sequencing, isolated DNA was measured via Spectrophotometer ND‐1000 (NanoDrop). DNA libraries were prepared using the Illumina TruSeq Nano protocol (Illumina, San Diego, CA, USA). Sequencing was performed on the Illumina MiSeq system (Illumina, San Diego, CA, USA) with a run configuration of paired‐end (PE) reads at 2 × 250 bp. Genomic analyses were conducted at the Functional Genomics Center Zurich (FGCZ) of the UZH and ETH Zurich. The sequencing reads were trimmed and filtered using CLC Genomics Workbench v11.0 (QIAGEN CLC bio, Aarhus, Denmark). Prophage sequences were identified in silico using PHASTEST (PHAge Search Tool with Enhanced Sequence Translation) (Wishart et al. 2023).

DNA enrichment and exclusion analysis was conducted using coverage data derived from BAM files aligned to the reference genome [*V. cholerae* O1 biovar El Tor str. N16961, Chromosomes 1 and 2 (NCBI 2024a, NCBI 2024b, Heidelberg et al. 2000)]. Genomic regions were divided into overlapping windows of 1000 bp with a step size of 100 bp using a custom script and saved in the Browser Extensible Data (BED) file format. Coverage for each region was calculated using ‘bedtools multicov’ (Quinlan and Hall 2010) with the resulting data saved as individual files for each sample. Further statistical analysis was performed with a custom Python script calculating the average read counts, log_2_ fold changes, *Z* scores, and *p* values, followed by Benjamini–Hochberg false discovery rate (FDR) correction to identify statistically significant over‐ or under‐represented regions (*p* values  ≤ 0.05).

In addition, the GC content for each defined region was calculated using sequences extracted from the reference genome with ‘bedtools getfasta’ (Quinlan and Hall 2010). A custom Python script generated scatter plots of GC content versus the log_2_ fold change for different genomic sections, including the base genome, CTX‐Phage and ICP‐Phage regions. Pearson correlation coefficients (*r*) and *p* values were calculated for each section to evaluate the relationship between GC content and enrichment.

Motif searches for genes controlled by transcriptional regulators of virulence [ToxR (AAAAAAMATMAAA; M signifying an amine; A or C) (Miller et al. 1987), ToxT (TTTTGAT) (Kazi et al. 2016), and TcpP (TGTAA‐N(6)‐TGTAA) (Goss et al. 2010)] were performed using the FIMO tool from the MEME Suite (Bailey et al. 2015; Bailey and Elkan 1994) via the generation of custom motif files in the MEME format. The search was conducted with an adjusted *p* value  ≤ 0.001 cutoff to accommodate shorter motif lengths.

### 2.13 Rhodamine Staining of BEVs

BEVs were labelled with rhodamine for subsequent internalisation studies as described previously with minor modifications (Zingl et al. 2021; Thapa et al. 2023). Five‐hundred micrograms protein equivalent of isolated BEVs were diluted in staining buffer [50  mM Na_2_CO_3_, 100 mM NaCl (pH 9.2)] to a final volume of 1 mL. After the addition of 100 µL octadecyl rhodamine B chloride (R18) (Thermo Fisher) to a final concentration of 0.5 mg mL^−1^, BEVs were stained in the dark with constant agitation O/N at room temperature. Finally, R18‐labelled BEVs were subjected to density gradient purification as described previously (Roier et al. 2016). Afterwards, the protein amount of the BEVs was measured via Bradford.

### 2.14 Internalisation of BEVs in Bacterial Cells

A modified version of the uptake assays previously established to monitor internalisation of BEVs in eukaryotic host cells was used (Zingl et al. 2021; Thapa et al. 2023; Bomberger et al. 2009; Kunsmann et al. 2015). O/N cultures of the respective recipient strains were grown in SOC: HEPES and diluted to a starting OD_600_ of 0.02 in SOC: HEPES. After adding rhodamine‐stained BEVs to a final concentration of 10 µg mL^−1^, 100 µL were transferred into 96‐well plates and the fluorescence (excitation: 554/emission: 590 nm) was measured every 30 min for 20 h using a microplate reader (FLUOstar Omega, BMG LabTech). To avoid precipitation of cells the plate was subjected to 300 rpm shaking between the readings. Readings using wells with SOC: HEPES and BEVs but no bacterial cells served as controls to monitor the background fluorescence as well as natural dequenching of rhodamine fluorescence over time and were used to correct the test sample values by subtraction.

### 2.15 Competition Assays

Competition assays in adult mice (in vivo) or LB (in vitro) were performed with a 1:1 mixtures of a respective mutant strain containing a functional *lacZ* gene and an isogenic *V. cholerae* WT strain (*lacZ^−^*) to allow differentiation between the strains on X‐Gal plates as previously described (Zingl et al. 2020; Pressler et al. 2016). All animal experiments were conducted in accordance with the rules of the ethics committee at the University of Graz and the corresponding animal protocols, which have been approved by the Austrian Federal Ministry of Science and Research Ref. II/10b (39/12/75ex2017/18 and 39/5‐4/75ex2023/24). The mice were housed with ad libitum access to food and water and monitored under the care of full‐time staff.

For evaluation of the in vivo colonisation fitness, we used the adult mouse model established by Nygren et al., which results in a stable colonisation of the cecum and colon (Nygren et al. 2009; Seper et al. 2013). Briefly, 6–8‐week old C57BL/6 mice were given Sm (5 mg mL^−1^) in their drinking water, 2 days before infection and kept on Sm‐water (0.2 mg mL^−1^) after inoculation with the respective *V. cholerae* mixture. Mice were infected by oral gavage with a 1:1 mixture of the mutant strain of interest and the isogenic WT strain (*lacZ^−^*), administering 7–10 × 10^10^ CFU per mouse. Appropriate dilutions of the inoculum were plated on LB‐Sm/X‐Gal plates to determine the exact input ratio. Approximately 48 h post‐infection mice were euthanised, their colons were removed and homogenised in 1 mL of LB with 20% glycerol. In vitro competitions in LB were performed in parallel by inoculation of 2 mL liquid culture with ∼10^5^ CFU from the inoculum and subsequent cultivation for ∼22 h at 37°C and 180 rpm. CFUs were determined by plating appropriate dilutions of the homogenised intestine or culture grown in vitro on LB‐Sm/X‐Gal plates. Results are given by the competition index (CI), which is the ratio of *lacZ* ^+^‐CFU to *lacZ* ^−^‐CFU normalised for the input ratio.

### 2.16 Horizontal Gene Transfer (HGT) Assays

BEVs isolated from VC1620/1::*tetR* (BEVs^VC1620/1::^ *^tetR^*) along with diverse recipients, that is, WT, Δ*comEA*, Δ*tfoX* and Δ*pilA* were used for HGT assays. To remove non‐luminal DNA 1.5–3.0 × 10^11^ BEVs^VC1620/1::^ *^tetR^* were mixed with 50 µL DNase buffer (10x) and incubated with 3 µL of recombinant DNase I (RNase‐free, 10 U µL^−1^, Roche) for 10 min at 37°C followed by heat inactivation at 80°C for 25 min, and stored at −80°C to be used within a week. In case of additional proteolytic digest, trypsin (Sigma–Aldrich, 30 µg mL^−1^ final concentration) and CaCl_2_ (10 mM final concentration) were added directly after the DNase reaction and incubated for 20 min at 55°C followed by heat inactivation as described above. Absence of living cells in BEVs^VC1620/1::^ *^tetR^* used for HGT was routinely confirmed by plating amounts equal to those used in the HGT assays on LB plates, which did not yield in colony formation after O/N incubation.

In case of in vitro assays, O/N cultures of the recipient bacterial strain grown in LB were diluted (1:100) in LB, AKI or SOC: HEPES and grown for 4 h anaerobically, followed by 4 h with aeration (180 rpm). Subsequently, the cultures were diluted in fresh LB, AKI or SOC: HEPES to a defined OD_600_ and 15 mL of the recipient suspension was mixed with BEVs^VC1620/1::^ *^tetR^*. The exact OD_600_ of the recipient as well as the amount of BEVs^VC1620/1::^ *^tetR^ varied* along the assays, but is indicated for each assay in the respective figure legend. 100 ng of purified, PstI‐digested pBR322 (linearised), which represents at least two‐fold higher DNA amounts compared to the highest BEVs^VC1620/1::^ *^tetR^* concentration used, served as control to exclude interference by the uptake of free DNA via natural competence. Linearised pBR322 serving as control for HGT assays was generated by digestion of purified pBR322 with PstI (20 U µL^−1^, NEB) according to the manufacturer protocol and subsequently heat inactivated at 80°C for 25 min. A proportion of the digestion reaction was analysed by agarose gel electrophoresis alongside undigested purified plasmid as control to conform complete linearisation. After the addition of BEVs^VC1620/1::^ *^tetR^* or linearised pBR322 to the recipient, the suspension was incubated for 20 h at 30°C and 150 rpm. Subsequently, cells were harvested by centrifugation (4000 x *g* for 10 min at RT) and resuspended in 1 mL LB. Total CFU was determined by plating appropriate dilutions LB‐Sm plates, while 100 µL aliquots of the residual suspension were plated on LB‐Tet plates to select for transformants. Tet^R^ colonies were streaked on LB‐Sm to control for Sm^R^, and presence of the *tetR*‐cassette was verified by PCR. The transformation rate was calculated as follows: Tet^R^ CFU/Sm^R^ CFU.

In general, the same protocol was used if BEVs isolated from VC1620/1::*cmR* (BEVs^VC1620/1::^ *^cmR^*) cultivated in virulence inducing (AKI) condition in presence or absence of bile were used as donor material in HGT assays with the following modifications: After incubation with recipient cells, 100 µL aliquots of the residual suspension were plated on LB‐Cm plates to select for transformants. Total CFU was determined by plating appropriate dilutions LB‐Sm plates. Cm^R^ colonies were streaked on LB‐Sm to control for Sm^R^ and presence of the *cmR*‐cassette was verified by PCR.

In case of in vivo assays, 6–8‐week old C57BL/6 mice were given Sm (5 mg mL^−1^) in their drinking water 2 days prior and kept on Sm‐water (0.2 mg mL^−1^) after inoculation with the respective *V. cholerae* strain as described above (see ‘Competition assays’). Mice were infected by oral gavage with the isogenic WT strain or with *∆comEA* administering 7–10 × 10^10^ CFU per mouse. Four to five faecal pellets were collected directly before infection as well as 24 h post‐infection, resuspended in 1 mL LB and plated on LB‐Tet plates to ensure the absence of Tet^R^ colonies at this time points as well as on LB‐Sm plates to confirm effective colonisation after infections. On Day 1 and Day 2 post‐infection, mice were orally gavaged twice a day with 100 µL BEVs^VC1620/1::^ *^tetR^* (330 µg protein equivalent, corresponding to approximately 2.5 × 10^11^ particles). On Day 4 post‐infection, mice were euthanised, and their colons were removed and homogenised in 1 mL of LB with 20% glycerol. Total CFU was determined by plating appropriate dilutions of the homogenised colon on LB‐Sm plates, while 100 µL aliquots of homogenised colon and appropriate dilutions were plated on LB‐Tet plates to select for transformants. Tet^R^ colonies were streaked on LB‐Sm to control for Sm^R^ and the presence of the TetR‐cassette was verified by PCR. The transformation rate was calculated as follows: Tet^R^ CFU/Sm^R^ CFU.

## 3 Results

### 3.1 Isolation and Characterisation of Stress‐Induced BEVs

**FIGURE 1:**
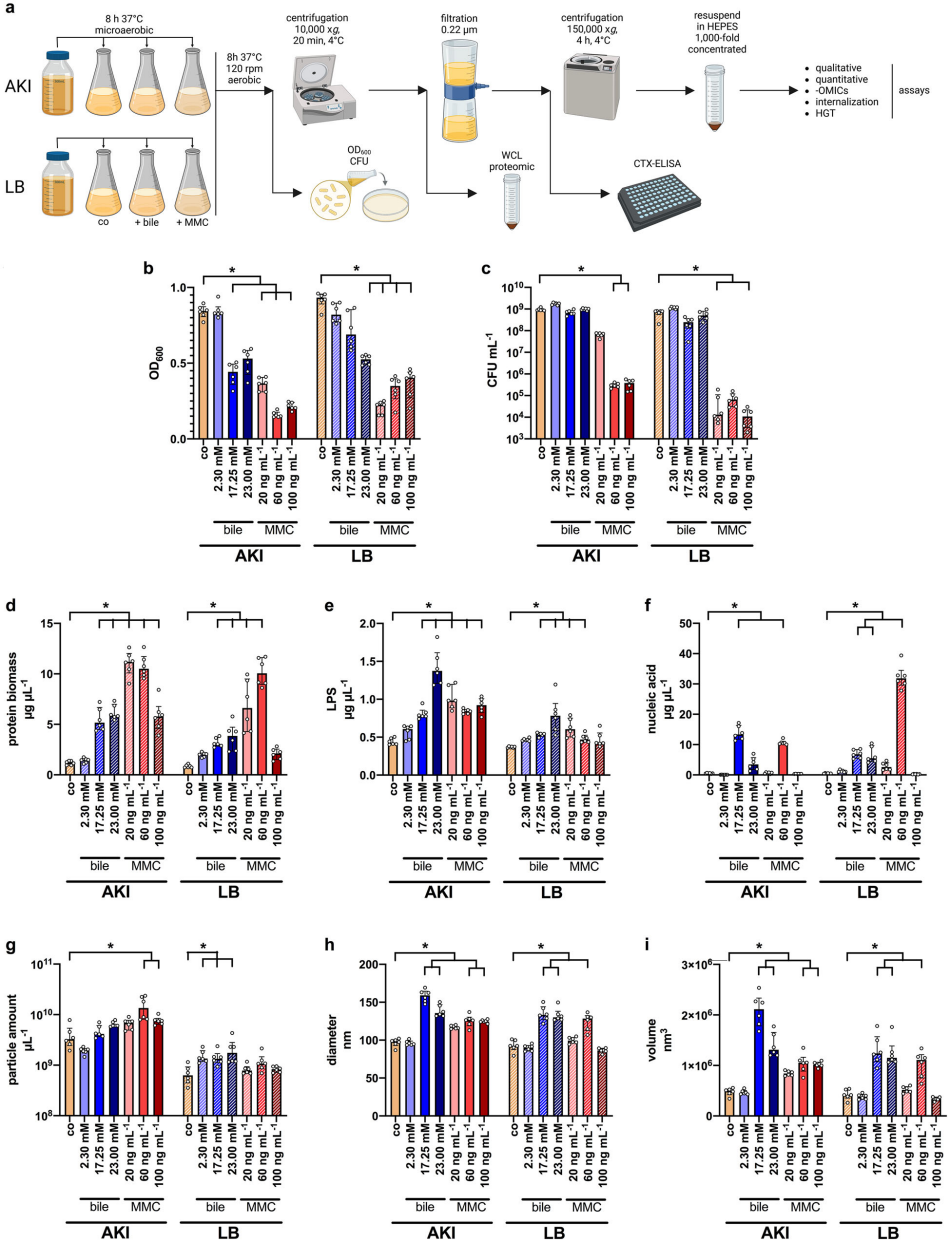
Isolation and characterisation of BEVs derived from control and stressor‐exposed *V. cholerae* cultures. (a) Schematic workflow of BEV isolation from *V. cholerae* cultivated under control and stress‐induced conditions. *V. cholerae* was grown microaerophilic in virulence (AKI) and non‐virulence inducing conditions (LB) and subsequently subjected outgrowth with aeration in presence (bile or mitomycin C (MMC) or absence (control, co) of stressors as indicated (see methods for detail). At the endpoint, growth parameters (OD_600_ and CFU) were assessed and BEVs were isolated from culture supernatants by centrifugation and filtration steps. WCLs for proteomic analysis were collected from the samples before filtration. Finally, BEV samples were resuspended in HEPES buffer (10 mM, pH 7.4) and subjected to downstream analyses. Created in BioRender. Fleischhacker (2025) https://BioRender.com/miw8w1c. (b, c) Impact of bile and MMC on the growth of *V. cholerae* cultures used for BEV isolation. OD_600_ and CFU were assessed after growth in virulence (AKI) and non‐virulence inducing conditions (LB) in absence (control, co) or presence of stressors (bile or MMC) at the indicated concentrations. (d) Total protein biomass of the BEV preparations determined by Bradford. (e) LPS amount of the BEV preparations determined by purpald assay. (f) Nucleic acid amount of the BEV preparations determined by SYTO‐9 staining using isolated chromosomal DNA of *V. cholerae* as standard. (g) Nanoparticle amount in the BEV preparations determined by nanoparticle tracking analysis (NTA). (h) Mean nanoparticle diameters of the BEV preparations determined by ZetaSizer Nano ZS90. (i) Volume of the BEVs calculated from the mean nanoparticle diameters determined via ZetaSizer Nano ZS90. (b–i) Data are presented as median ± interquartile range (IQR). For the AKI and LB data sets significant differences between the control and stress‐induced samples were analysed via a Kruskal–Wallis test with uncorrected Dunn's multiple comparison (*p < 0.05, *n* = 6). BEV, bacterial extracellular vesicle; WCL, whole cell lysate.

To investigate stress‐induced vesiculation, we established a pipeline for the isolation of BEVs from *V. cholerae* cultures grown under virulence inducing conditions (AKI) and non‐virulence inducing conditions (LB) with and without exposure to three different doses of bile or MMC (Figure 1a). As the AKI virulence induction protocol requires a microaerophilic cultivation followed by outgrowth with aeration, we applied the same procedure to the non‐virulence inducing LB cultures. This design enabled the preparation of comparable BEV samples from cultures with only one parameter changed at a time: cultivation in AKI versus LB, exposure to bile or MMC or no stressor as control. All other conditions, including growth durations, temperature and aeration, were kept identical. The three MMC doses were selected based on published protocols for the induction of explosive vesicles in other bacteria (Turnbull et al. 2016; Toyofuku et al. 2017; Andreoni et al. 2019). The three bile concentrations were selected to reflect the physiological range in the human gut. Although the quantity and composition of bile salts are highly dependent on the fastening status and diet, intestinal concentrations in humans have been reported to range between 3.6 and 24.0 mM (Bergström et al. 2014).

The impact of bile and MMC on growth behaviour was monitored by OD_600_ measurements as well as CFU quantification the time point of harvest (Figure 1b, c). In general, all MMC as well as higher bile concentrations resulted in lower OD_600_ values, indicating cellular stress in both AKI and LB cultures. A reduction in CFU was observed only in the presence of MMC, which might reflect the distinct modes of action of the two stressors, with MMC being a DNA intercalating substance and bile acting as a membrane emulsifier. Moreover, a hallmark of *V. cholerae* is a relatively high bile tolerance combined with efficient bile adaptation mechanisms (Klose 2001; Simonet et al. 2003; Bina et al. 2018; Cerda‐Maira et al. 2008), which may explain the lack of a significant CFU decrease under bile exposure. BEVs were isolated from the differential cultures by routine filtration and centrifugation steps established in the laboratory and were subjected to various assays to assess quantity and quality parameters, including protein, LPS and nucleic acid biomass, as well as particle number, diameter and volume (Figure 1d–i). In general, the BEVs from cultures exposed to bile or MMC showed an increase in all measured parameters compared to the control BEVs from unstressed cultures, while the magnitude of these increases varied across parameters. Exposure to bile or MMC increased protein biomass of the BEV samples up to 10‐fold compared to the control, while LPS levels increased only two–three‐fold. Most notably, nucleic acids were readily detected in BEVs from stress exposed cultures, while control BEVs contained only traces of them that were barely detectable. This indicates the presence of cytosolic material in BEVs released under stress. The strongest effect was observed with 17.25 mM bile and 60 ng mL^−1^ MMC, which resulted in up to 60‐fold higher nucleic acid levels compared to control BEVs. Both lower and higher stressor doses resulted in less nucleic acid loading, suggesting that optimal stressor concentrations exist for maximal nucleic acids incorporation into BEVs. BEV amounts and diameter also increased upon exposure to stressors. While BEV counts increased by up to five‐fold, the rise in average BEV diameters was particularly striking, yielding much larger BEVs with substantially greater luminal volume (Figure 1h, i). Since the protein biomass and the nucleic acid content in stress‐induced BEVs increased more than their absolute numbers, a relative enrichment of these components was also evident after normalisation to the particle number (Figure S1). Based on these results, 17.25 mM bile and 60 ng mL^−1^ MMC were selected as stressor doses for the remainder of the study, as they resulted in a significant increase in protein biomass and nucleic acids content in BEVs under virulence inducing and non‐virulence inducing conditions (Figure 1d, f).

The selected BEV preparations were further characterised by TEM analyses, which enabled visualisation of spherical particles of variable sizes (Figure S2). Consistent with the particle size determination by ZetaSizer measurements (Figure 1h), TEM images revealed an overall larger diameter of BEVs derived from bile‐ or MMC‐exposed cultures compared to control BEVs (Figure S2). All BEV preparations contained spherical‐shaped vesicles with no evidence of substantial contamination by bacterial flagellar‐ and pili‐like structures. Notably, phage‐like structures could be readily detected in BEV preparations from MMC‐exposed cultures, but were absent from BEVs derived from bile‐exposed cultures or control cultures. This observation is consistent with the role of MMC as a well‐documented activator of the bacterial SOS response and prophage induction (Turnbull et al. 2016; Toyofuku et al. 2017; Baharoglu and Mazel 2011; Otsuji et al. 1959; Mohanraj and Mandal 2022; Wagner et al. 2002; Owen et al. 2017).

In order to evaluate whether the purity of the original BEVs could be improved by an additional purification step, defined protein equivalents of the original BEV preparations were subjected to density‐gradient purification, after which the protein and nucleic acid concentrations in the individual fractions were assessed quantitatively (see Figure S3). Along the harvest of the individual fractions, a single distinct, opaque band corresponding to Fraction 2 was present in all BEV preparations. Consistently, only Fraction 2 contained substantial amounts of proteins and nucleic acids, while all other fractions exhibited signal intensities below or close to the detection limit. Fraction 2 of each density gradient purification was also subjected to nanoparticle tracking analysis (NTA) and DLS (Zetasizer) to quantify particle concentrations and assess size distributions. Comparative analysis of the original BEV preparations and the respective gradient‐purified BEVs revealed similar mode diameters, indicating that the predominant vesicle populations remained essentially unchanged. To assess potential morphological alterations, TEM was performed on density‐gradient purified BEVs derived from bile‐ or MMC‐exposed cultures (Figure S4). TEM imaging of the gradient‐purified preparations revealed no obvious structural changes compared to the corresponding original BEV preparations (Figures S2 and S4). Moreover, the continued presence of phage‐like structures in density‐gradient purified BEVs from MMC‐exposed cultures suggests that the density‐gradient purification is insufficient to eliminate such aggregates from BEV preparations. As the density‐gradient step did not improve the BEV purity but may impose further physical and artificial stress on the BEVs, the density‐gradient was omitted and original BEV preparations were used for all further experiments.

**FIGURE 2:**
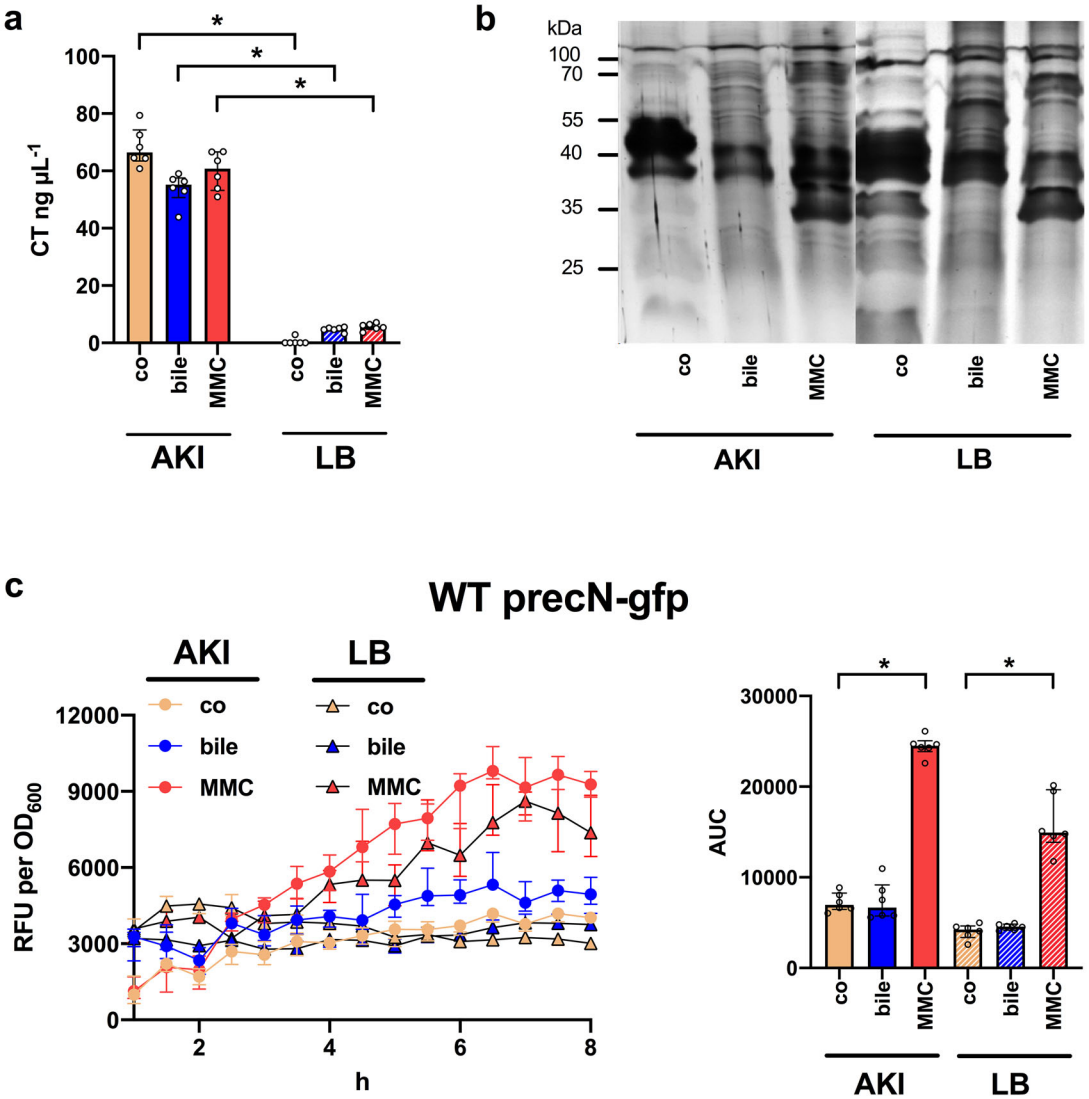
Impact of stressors on virulence activation, BEV protein composition and SOS induction. (a) Total amount of cholera toxin (CT) in the sterile filtered supernatants of cultures grown under virulence (AKI) and non‐virulence inducing conditions (LB) in presence of bile (17.25 mM) or MMC (60 ng mL^−1^) or without any stressor (control, co). (b) Shown are protein profiles of BEVs isolated from *V. cholerae* WT cultivated in virulence (AKI) and non‐virulence inducing conditions (LB) presence of bile (17.25 mM) or MMC (60 ng mL^−1^) or absence of stressors (control, co). Approximately 3 µg protein equivalent of each sample was loaded onto the gels, separated by SDS‐PAGE and protein bands were visualised by silver staining. Lines to the left indicate the molecular masses of the protein standards in kDa. (c) Induction of the SOS response assessed by the GFP fluorescence measured in the WT precN‐gfp reporter strain cultivated in virulence (AKI) and non‐virulence inducing conditions (LB) presence of bile (17.25 mM) or MMC (60 ng mL^−1^) or without any stressor (control, co). The data is presented as median relative fluorescence units (RFU) divided by the OD_600_ measured in parallel every 30 min. The bar chart on the right shows the median area under the curve (AUC) ± IQR calculated from the RFU assays. (a, c) Data are presented as median ± IQR. Statistical differences between the control and stress‐induced samples were analysed via a Kruskal–Wallis test with uncorrected Dunn's multiple comparison (*p < 0.05, *n* = 6). BEV, bacterial extracellular vesicle; GFP, green fluorescent protein; IQR, interquartile range; MMC, mitomycin C; SDS‐PAGE, sodium dodecyl sulphate‐polyacrylamide gel electrophoresis.

To assess whether stressors impact on virulence induction, cholera toxin amounts were quantified by ELISA (Figure 2a). Neither bile nor MMC exposure altered cholera toxin expression during AKI cultivation compared to the respective control without stressor exposure. In contrast, both stressors caused slightly, though not statistically significant, increase in cholera toxin production in LB. Comparative protein profiling of BEVs isolated from bile or MMC exposed cultures (BEV^AKI‐bile^, BEV^AKI‐MMC^, BEV^LB‐bile^ and BEV^LB‐MMC^) compared to the respective control BEVs (BEV^AKI‐co^ and BEV^LB‐co^) identified distinct proteomic alterations (Figure 2b). This suggests that not only stressor‐induced BEVs differ in protein composition from control BEVs but also that bile‐induced BEVs and MMC‐induced BEVs exhibit stressor‐specific changes. While MMC is well‐known to induce genotoxic stress and SOS response (Turnbull et al. 2016; Toyofuku et al. 2017; Baharoglu and Mazel 2011), data on bile remain scarce. Induction of SOS response upon exposure to bile or MMC in AKI and LB cultivation was assessed using a reporter construct carrying the GFP fused to the *recN* promoter, an established SOS response marker in *V. cholerae* (Baharoglu and Mazel 2011). MMC exposure resulted in a significant increase in GFP expression, indicating SOS induction, while bile exposure had no effect compared to the control condition (Figure 2c). Thus, MMC and bile trigger different cellular stress responses in *V. cholerae*, both leading to stress‐induced BEV release.

### 3.2 Stress‐Induced BEVs Exhibit Distinct Changes in Protein Composition

**FIGURE 3:**
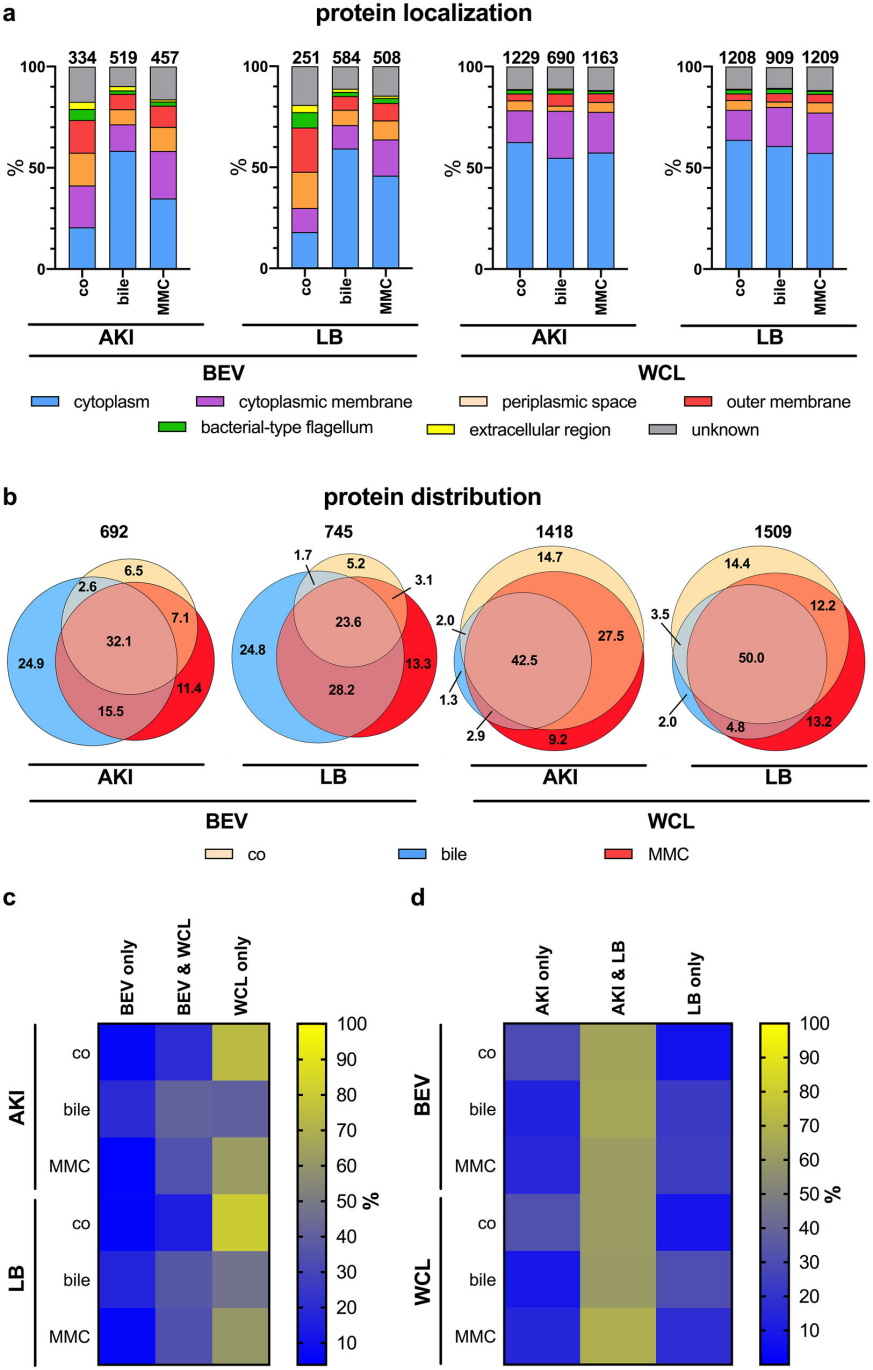
Proteomic analyses of BEVs and whole cell lysates (WCLs) derived from control and stressor‐exposed *V. cholerae* cultures. (a) Stacked bar charts displaying the percentage distribution of the predicted cellular localisation for the proteins identified in the BEV preparations or corresponding WCL. BEVs and WCL were obtained from *V. cholerae* WT cultivated in virulence (AKI) and non‐virulence inducing conditions (LB) presence of bile (17.25 mM) or MMC (60 ng mL^−1^) or without any stressor (control, co). The total number of identified proteins is given at the top of each bar. (b) Venn diagram visualising the overlap of the identified proteins in the BEV preparations or WCL obtained from *V. cholerae* WT cultivated in virulence (AKI) and non‐virulence inducing conditions (LB) in presence of bile (17.25 mM) or MMC (60 ng mL^−1^) or without any stressor (control, co). (c) Heatmap visualising the percentage distribution of unique or common proteins identified in the BEV preparation and its corresponding WCL. For each of the differential growth condition (indicated by the rows), the colour indicates the percentage of proteins identified only in the BEV preparation (left column), in the BEV preparation as well as in the corresponding WCL (middle column) or proteins identified only in the WCL (right column). (d) Heatmap visualising the percentage distribution of unique or common proteins identified in samples derived from virulence (AKI) and non‐virulence inducing conditions (LB). For each of the differential BEV preparation or WCL (indicated by the rows), the colour indicates the percentage of proteins identified only in samples derived from AKI cultures (left column), in samples derived from AKI as well as LB cultures (middle column) or proteins identified only in samples derived from LB cultures (right column). (a–d) LC‐MS/MS analysis of three biological replicates (*n* = 3). Proteins were included if detected in ≥2 of 3 replicates for the respective condition. BEV, bacterial extracellular vesicle; MMC, mitomycin C.

Intrigued by the differential protein profiles, we analysed the proteome of the differential BEVs and corresponding whole cell lysates (WCLs) derived from *V. cholerae* cultivated in AKI or LB medium, with or without exposure to bile or MMC. This comprised six BEV types (BEV^AKI‐bile^, BEV^AKI‐MMC^, BEV^AKI‐co^, BEV^LB‐bile^, BEV^LB‐MMC^ and BEV^LB‐co^) and their corresponding WCLs (WCL^AKI‐bile^, WCL^AKI‐MMC^, WCL^AKI‐co^, WCL^LB‐bile^, WCL^LB‐MMC^ and WCL^LB‐co^). Proteomes were analysed by LC‐MS/MS using three biological replicates of each BEV and WCL type (Figure 3, Table S3). Using a cutoff of ≥2 predicted peptides protein, we identified 251–584 proteins in BEV preparations and 690–1229 proteins in WCLs. Among BEVs, the lowest numbers were observed in the control samples (334 for BEV^AKI‐co^ and 251 for BEV^LB‐co^), whereas stress‐induced BEVs contained nearly twice as many proteins. As expected, the highest protein counts were detected in WCLs with more than 1000 in controls and MMC‐exposed WCLs and slightly fewer in bile‐exposed WCLs. Predicted protein localisation was analysed in silico using UniProt and PSORTb v3.0 (UniProt Consortium 2023; Yu et al. 2010) (Figure 3a). WCLs consistently showed a similar distribution, with most proteins localised to the cytoplasm or cytoplasmic membrane (78%–80%). Consistent with previous BEV proteomics studies in Gram‐negative bacteria (Toyofuku et al. 2023; Altindis et al. 2014; Roier et al. 2015; Bhar et al. 2021; Juodeikis et al. 2024; Lappann et al. 2013), control BEVs (BEV^AKI‐co^ and BEV^LB‐co^) were enriched in outer membrane (16%–22%), periplasmic (16%–18%), flagellar (5%–8%) and extracellular proteins (4%). In contrast, bile‐ and MMC‐induced BEVs mainly contained cytoplasmic or cytoplasmic membrane proteins (58%–71%), regardless of culture condition. Enrichment analyses of shared proteins across BEV preparations confirmed the shift: stress‐induced BEVs showed log_10_ enrichment values >1 for cytoplasmic and cytoplasmic membrane proteins, while outer membrane, periplasmic and flagellar proteins were reduced (log_10_ values <1) (Figure S5a). Notably, bile‐ and MMC‐induced BEVs showed similar shared protein patterns under both AKI and LB conditions, reflected by enrichment values close to 0. Comparison between BEVs and their corresponding WCLs further revealed that stress‐induced BEVs and WCLs become more similar in protein composition, with enrichment values close to 0 (Figure S5b). In contrast, control BEVs and WCLs exhibited greater divergence. WCLs from AKI or LB cultures, whether stressed or not, displayed only minor differences and much less variation than BEVs (Figure S5c). Cultivation conditions (AKI vs. LB) thus had little impact on enrichment patterns for either BEVs or WCLs (Figure S5d). In summary, compared to control BEVs the bile‐ and MMC‐induced BEVs are characterised by greater protein diversity and a protein localisation pattern more closely resembling WCLs. This supports current models of stress‐induced BEV biogenesis via cell lysis and vesicle self‐assembly of from shattered membrane fragments, enabling incorporation of cytoplasmic components (Toyofuku et al. 2019; Toyofuku et al. 2023; Turnbull et al. 2016; Ebenberger et al. 2024; Toyofuku et al. 2017).

We next assessed the overlap of proteins in control and stress‐induced BEVs and WCL derived from AKI and LB cultures (Figure 3b). The core BEV proteome shared by control and stress‐induced BEVs accounted for 32.1% (AKI) and 23.6% (LB), respectively. In addition, bile‐ and MMC‐induced BEVs shared 15.5% (AKI) or 28.2% (LB) proteins not present in the respective control condition, likely representing a common BEV stress‐proteome. For both cultivation conditions, bile‐induced and MMC‐induced BEVs also contained a substantial sets of unique proteins found exclusively in the individual preparations (i.e., BEV^bile^ or BEV^MMC^). In comparison, control and stress‐induced WCLs exhibit a much larger core proteome with 42.5% (AKI) or 50.0% (LB) overlap. Conversely, the stress‐proteome defined as the overlap of proteins between bile‐ and MMC‐induced WCL is less than 5% under both cultivation conditions and is thus much smaller than that of BEVs.

The distribution of unique and shared proteins between BEVs and their corresponding WCLs was also analysed for each of the six differential growth conditions (Figure 3c). Under control conditions without stressors, BEVs and WCL shared only a minor fraction of proteins. However, in the presence of bile or MMC the proportion of shared proteins increased, reinforcing the observation that stress‐induced BEVs are enriched in cytoplasmic and cytoplasmic membrane proteins typically found in WCLs but largely absent in control BEVs. Finally, we examined the cultivation‐dependent distribution of unique and shared proteins in control and stress‐induced BEVs and WCLs (Figure 3d). In general, AKI‐ and LB‐derived control BEVs or the corresponding WCLs share about 75% of proteins, and exposure to stressors had only a minor effect on this distribution.

Unlike the SOS response‐inducing agent MMC, bile is a previously uncharacterised stressor for BEV induction. Thus, the proteomes of density‐gradient purified, bile‐induced BEVs generated under both virulence (AKI) and non‐inducing conditions (LB) were also analysed by LC‐MS/MS using three biological replicates (Figure S6 and Table S3). The density‐gradient purified BEVs exhibited a similar distribution of protein localisation to the original bile‐induced BEVs, with cytoplasmic and cytoplasmic membrane proteins comprising the majority of the detected proteins (Figure 3a and Figure S6a). Further quantitative analyses corroborated this similarity, revealing no substantial differences between the original and density‐gradient purified BEVs, except for a significant reduction of ‘bacterial‐type flagellum’ components in the latter (Figure S6b). However, flagellar components are not expected to impact the downstream assays provided in this study. In line with density‐gradient fraction characterisation (Figures S3 and S4), proteome analysis shows that the additional density‐gradient purification has only a marginal benefit on the BEV purity.

To further characterise the cellular stress responses triggered by bile and MMC, we performed a quantitative analysis of the WCL MS data focusing on proteins associated with previously described SOS regulons (Table S4). Of the 37 SOS‐regulated genes identified in a previous report (Krin et al. 2018), 23 corresponding proteins were detected in the WCLs analysed in this study. Bile exposure elicited a comparatively modest response, with increased abundance (≥2‐fold) observed for 5 out of 23 proteins under virulence inducing conditions (AKI) and 2 out of 23 proteins in non‐virulence inducing conditions (LB). In contrast, MMC treatment resulted in a substantially higher presence of SOS‐regulated proteins, with 12 out of 23 proteins increased in AKI and 14 out of 23 in LB cultures. Consistent with our GFP‐based reporter assays (Figure 2c), RecN protein levels increased markedly upon MMC exposure but remained unchanged under bile treatment (Table S4). Notably, some SOS‐associated proteins also showed reduced expression (≤2‐fold) in presence of stressors, pointing to a complex and condition‐dependent regulatory response.

### 3.3 Stress‐Induced BEVs Contain Genomic DNA

**FIGURE 4:**
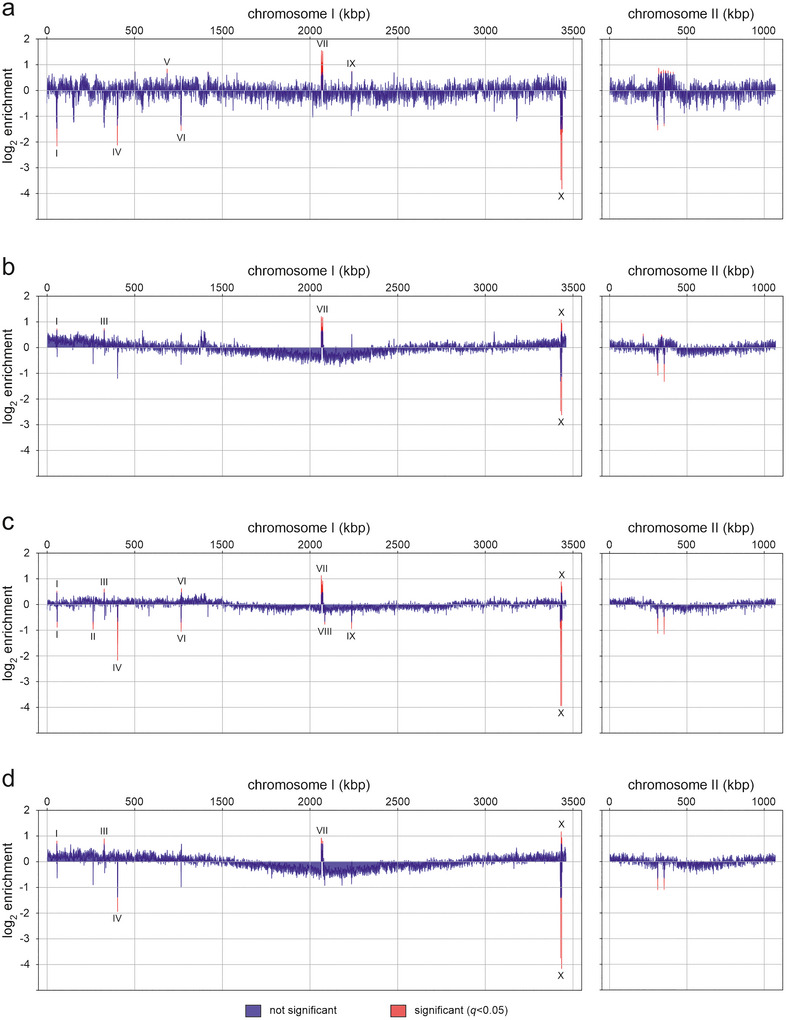
Log_2_ enrichment of kbp partitions of the *V. cholerae* Chromosomes 1 and 2 identified in stress‐induced BEVs. Shown are the relative abundancies of chromosomal regions (left: Chromosome I; right: Chromosome II) in the luminal DNA of BEVs identified by sequencing. BEVs were isolated from *V. cholerae* WT cultivated in virulence (AKI) and non‐virulence inducing conditions (LB) in the presence of bile (17.25 mM) or MMC (60 ng mL^−1^), corresponding to AKI‐bile (A), AKI‐MMC (B), LB‐bile (C) and LB‐MMC (D). Differential abundancies of chromosomal regions were calculated via the *Z* scores, and *p* values followed by Benjamini–Hochberg false discovery rate (FDR) correction red denoting statistically significant changes (*p* values ≤ 0.05). Areas with significant enrichment or depletion are highlighted as follows: (I) 23S rRNA (VCr002); (II) transposase OrfAB, subunit B (VC0257); (III) 23S ribosomal RNA (VCr008); (IV) 23S ribosomal RNA (VCr011); (V) initiation factor IF‐2 (VC0643); (VI) 23S ribosomal RNA (VCr014); (VII) CTXφ phage (1563500–1573600); (VIII) transposase OrfAB, subunit B (VC1478); (IX) *fraH* (VC1620); (X) cluster of multiple tRNA and rRNA in repeat (2927400–2938900). For each condition, BEVs derived from 6 to 8 independent biological replicates were pooled before DNA extraction and sequencing to ensure sufficient material for downstream analysis (resulting in the sequencing analysis of *n* = 1 per condition). BEV, bacterial extracellular vesicle; MMC, mitomycin C.

The DNA content of bile and MMC‐induced BEVs from both cultivation conditions was subjected to whole genome sequencing. Exterior DNA was removed by DNAse treatment prior to sequencing, which resulted in an almost complete loss of the already low nucleic acid amounts of the control BEVs. Consequently, control BEVs were excluded from sequencing analyses due to insufficient DNA yield. Sequencing of DNA from BEV^AKI‐bile^, BEV^AKI‐MMC^, BEV^LB‐bile^ and BEV^LB‐MMC^ yielded in approximately 2.7–150.2 M reads per sample. Given the genome size of *V. cholerae* (Chromosome 1: 2,961,149 bp; Chromosome 2: 1,072,315 bp), an equal distribution would correspond to 73.41% for Chromosome 1 and 26.59% for Chromosome 2 (Heidelberg et al. 2000). The median presence of Chromosome 1 ranged between 73.35% and 74.82%, excluding preferential packaging of one chromosome over the other. Mapped reads indicated variable representation of genomic regions in BEV‐associated DNA, with only ten regions of Chromosome 1 showed significant changes in at least one BEV type (Figure 4, Table S5). Regions I, III, IV, VI and X were located in rRNA and tRNA clusters, Regions II and VIII encoded hypothetical transposases, Region V encoded the initiation factor IF‐2 and Region VII contained the CTXφ prophage genes. Thus, these regions represent either essential genes or mobile elements. Most regions showed condition‐ and stressor‐dependent enrichment or exclusion, with one notable exception: Region VII (CTXφ), which showed the highest peak and was consistently enriched across all four BEV types. Region X showed inconsistent representation across samples with either very high or very low abundance and no clear pattern across BEV types. Because this region lies within a repetitive rRNA/tRNA cluster, this variability is likely influenced by limitations of the short‐read sequencing and mapping approach, which can complicate accurate read assignment in highly repetitive genomic regions and may therefore lead to artificial fluctuations in apparent coverage. Finally, Region IX was one of three regions significantly enriched in BEV^AKI‐bile^ and spanned ORF VC1620, which encodes the flagellum‐regulated hemagglutinin FrhA. Previous work by Syed et al. demonstrated that FrhA can act as an adhesion factors to epithelial cells or chitin and facilitates biofilm formation (Syed et al. 2009; Kitts et al. 2019).

With regard to GC‐content, BEV‐associated CTXφ DNA showed a negative correlation across all four all four BEV types (*R* values −0.24 to −0.42), whereas ICP phage DNA and the residual genome displayed no or only very weak correlations with condition‐ and stressor‐dependent variations (Figure S7). BEV‐associated DNA was also screened for differential abundance of DNA‐binding motifs for the key transcription factors involved in virulence regulation, that is, TcpP, ToxR and ToxT. However, none of the BEV types showed significant enrichment or depletion of these motifs.

Phage content was further analysed using the bioinformatic tool ‘PHASTEST’, which identified the filamentous bacteriophage CTXφ, located on Chromosome I (Positions 1564152–1577272), and fragments of the Bangladesh cholera phage 1 (ICP1), located on chromosome I (Positions 1925144–1948573) (Wishart et al. 2023; Heidelberg et al. 2000). The DNA content of all four stress‐induced BEVs (BEV^AKI‐bile^, BEV^AKI‐MMC^, BEV^LB‐bile^ and BEV^LB‐MMC^) was significantly enriched in CTXφ DNA (Figure 4, Region VII), whereas no enrichment or depletion was detected for the ICP1 phage DNA.

### 3.4 Stress‐Induced BEVs Mediate Horizontal Gene Transfer

Based on the presence of chromosomal DNA, the stress‐induced BEVs characterised here may contribute to HGT of chromosomal loci. As a proof‐of‐principle, we focused on bile and MMC‐induced BEVs derived from virulence inducing conditions, that is, BEV^AKI‐bile^ and BEV^AKI‐MMC^. To monitor BEV‐mediated HGT, we introduced a *tetR*‐cassette from the plasmid pBR322 into the intergenic region between VC1620 and VC1621, generating strain VC1620/1::*tetR*. This locus was chosen because it is significantly enriched in BEV^AKI‐bile^ (Figure 4), in silico analyses of the VC1620/21 region using PHASTEST and ISfinder found no evidence of mobile genetic elements, that is, phage‐related sequences or insertion sequences (Wishart et al. 2023; Siguier et al. 2006) and the *V. cholerae* El Tor reference genome from the KEGG database contains no annotations for essential genes in this region (Kanehisa et al. 2008; Kanehisa and Goto 2000; Kanehisa et al. 2006), consistent with the absence of any growth defect in the insertion mutant VC1620/1::*tetR* (see competition assays below).

In HGT experiments, *V. cholerae* WT served as the recipient, while DNase‐digested bile‐induced BEVs, MMC‐induced BEVs or control BEVs derived from VC1620/1::*tetR* were used as donor material. We tested variations in recipient cultivation conditions (LB, AKI and SOC: HEPES) and starting OD_600_ values (0.004, 0.2 and 0.8) (Figure S8a). Cultivation in the glucose containing ‘**S**uper **O**ptimal broth with a **C**atabolite repression’ SOC: HEPES medium resulted in median transformation rates significantly above the detection limit for all three starting OD_600_ values. SOC: HEPES is a nutrient rich broth frequently used for bacterial HGT experiments, as it promotes higher survival of transformants and thereby increases transformation efficiency (Sun et al. 2009; Yaron et al. 2000). Recipient cultivation in AKI resulted in detectable transformation rates for starting OD_600_ values of 0.004 and 0.2, while cultivation in LB supported detectable transformation only for starting OD_600_ values of 0.2. In summary, cultivation in SOC: HEPES medium and a recipient starting OD_600_ of 0.2 were identified as the most favourable conditions among those tested.

Using SOC: HEPES or AKI medium with a recipient starting OD_600_ of 0.2, we next tested the effect of varying BEV doses (Figure S8b). In SOC: HEPES, median transformation rates were significantly above the detection limit for all tested doses (1 × 10^10^, 2.5 × 10^11^, 1 × 10^12^), while AKI cultivation supported detectable transformation only at 2.5 × 10^11^ BEVs. Accordingly, a dose of 2.5 × 10^11^ was selected for all subsequent experiments.

**FIGURE 5:**
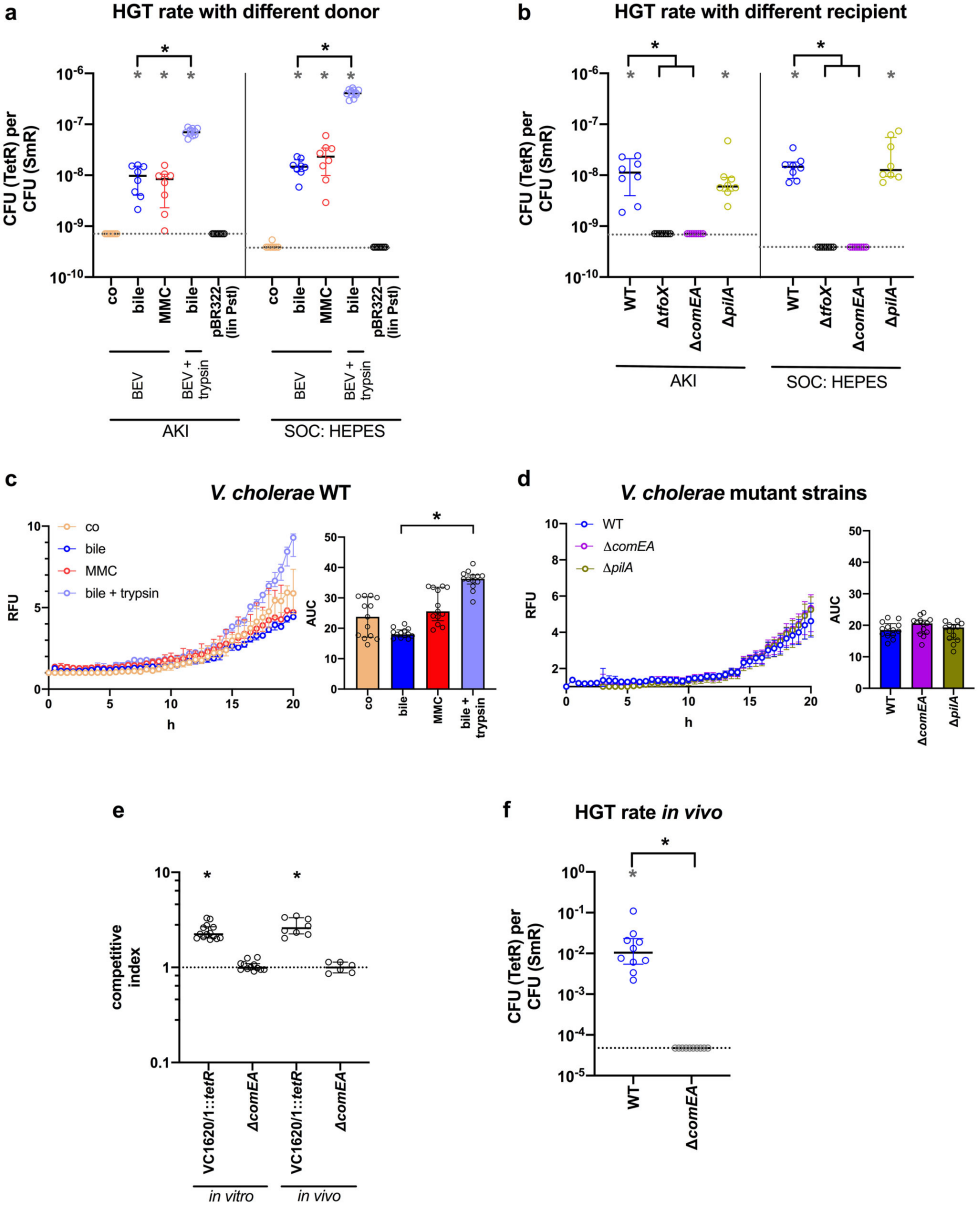
Stress‐induced BEVs are internalised by recipient cells and mediate horizontal gene transfer (HGT). (a) HGT rate of the *tetR*‐cassette using BEVs derived from VC1620/1::*tetR* (BEVs^VC1620/1::^ *^tetR^*) as donor. BEVs^VC1620/1::^ *^tetR^* were isolated from AKI cultures in presence of bile (17.25 mM) or MMC (60 ng mL^−1^) or without any stressor (control, co). In addition, trypsin digested BEVs^VC1620/1::^ *^tetR^* derived from AKI cultures in presence of bile (17.25 mM) were used (see methods for detail). The purified linearised plasmid pBR322 (lin PstI) served as an extracellular DNA control. *V. cholerae* WT grown in AKI or SOC: HEPES served as recipient and was incubated for 20 h at 30°C and 150 rpm with respective BEVs^VC1620/1::^ *^tetR^* (2.5 × 10^11^ particles) or pBR322 linearised with PstI (100 ng) before total CFU and transformants were determined by plating on LB‐Sm and LB‐Tet plates. (b) HGT rate of the *tetR*‐cassette using BEVs^VC1620/1::^ *^tetR^* derived from AKI cultures in presence of bile (17.25 mM) as donor and *V. cholerae* WT or diverse *V. cholerae* mutants as recipient. Recipients, grown in AKI or SOC: HEPES, were incubated for 20 h at 30°C and 150 rpm with respective BEVs^VC1620/1::^ *^tetR^* (2.5 × 10^11^ particles) before total CFU and transformants were determined by plating on LB‐Sm and LB‐Tet plates. (c) Internalisation of BEVs^VC1620/1::^ *^tetR^* by *V. cholerae* WT. Rhodamine‐labelled BEVs^VC1620/1::^ *^tetR^* derived from AKI cultures in presence of bile (17.25 mM) or MMC (60 ng mL^−1^) or without any stressor (control, co) were incubated with *V. cholerae* WT for 20 h. In addition, trypsin digested BEVs^VC1620/1::^ *^tetR^* derived from AKI cultures in presence of bile (17.25 mM) were used (see methods for detail). (d) Internalisation of BEVs^VC1620/1::^ *^tetR^* derived from AKI cultures in presence of bile (17.25 mM) by *V. cholerae* WT or the deletion mutants ∆*pilA* and ∆*comEA*. (c, d) Uptake is detected by an increase in relative fluorescence units (RFU) measured every 30 min. Wells containing rhodamine‐labelled BEVs^VC1620/1::^ *^tetR^* without recipient cells served as a blank. Shown is the median ± IQR (*n* = 13). The bar chart on the right shows the median area under the curve (AUC) ± IQR calculated from the internalisation assays. Asterisks highlight significant differences between respective data sets (*p < 0.05 Kruskal–Wallis test followed by post hoc Dunn's multiple comparisons). (e) Colonisation fitness of VC1620/1::*tetR* and ∆*comEA*. Results are shown as competitive index (CI) for competitions of VC1620/1::*tetR* or ∆*comEA* to a fully virulent LacZ^−^ derivative of the WT (*lacZ^−^*) in LB (in vitro) and in adult mice (in vivo). Each circle represents the CI from a single assay. Horizontal bars and error bars indicate the median ± IQR. Asterisks highlight data sets with significantly different CI from the theoretical value of 1 [*p* < 0.05, Wilcoxon test against a hypothetical value of 1, *n* = 14 for VC1620/21::*tetR*/WT (*lacZ^−^*) in vitro; *n* = 12 for ∆*comEA*/WT (*lacZ^−^*) in vitro; *n* = 8 for VC1620/21::*tetR*/WT (*lacZ^−^*) in vivo; and *n* = 6 for ∆*comEA*/WT (*lacZ^−^*) in vivo]. (f) In vivo HGT rate of the *tetR*‐cassette using BEVs^VC1620/1::^ *^tetR^* derived from AKI cultures in presence of bile (17.25 mM) as donor and *V. cholerae* WT or ∆*comEA* as recipient. Mice colonised with WT or ∆*comEA* received BEVs^VC1620/1::^ *^tetR^* VC1620/1::*tetR* by oral gavage twice a day for two days before mice were euthanised and total CFU and transformants were determined by plating the homogenised colon on LB‐Sm and LB‐Tet plates. (a, b, f) Data are presented as median ± IQR. Assays yielding in no transformants on LB‐Tet were set to limit of detection (LOD), which is indicated by a dotted line. The LOD was defined as 0.5 CFU detected in the highest concentration plated on LB‐Tet plates divided by the total CFU determined by plating on LB‐Sm plates. HGT rates significantly higher than the LOD were by evaluated by the Wilcoxon Signed Rank test against the hypothetical value of the LOD (*p < 0.05, *n* = 8 for Panels a and b, *n* = 10 for Panel f). Statistically significant differences between the bile‐induced BEVs^VC1620/1::^ *^tetR^* before and after trypsin digest (*n* = 8) or between the WT and ∆*comEA* colonised mice (*n* = 10) were analysed via the Mann–Whitney *U* test (*p < 0.05). BEV, bacterial extracellular vesicle; IQR, interquartile range; MMC, mitomycin C.

With these optimised conditions, side‐by‐side HGT assays were performed using MMC‐induced BEVs and control BEVs derived from non‐stressed cultures (Figure 5a). Transformation rates were comparable between bile‐ and MMC‐induced BEVs in both AKI and SOC: HEPES, indicating that BEV‐mediated HGT is independent of the stressor that triggers vesiculation.

To mimic the proteolytic environment of the intestine, bile‐induced BEVs were digested with trypsin before HGT assays. Trypsin digestion enhanced transformation rates by approximately 10‐fold (Figure 5a), suggesting that surface‐exposed proteins of the BEVs are not required, and may even hinder BEV interactions with bacterial recipient cells. In contrast, the addition of extracellular DNA, that is, the linearised plasmid pBR322, did not result in detectable transformants, despite being applied at 75‐fold higher DNA doses than those carried by stress‐induced BEVs. We chose linearised plasmid DNA (generated using PstI‐digested pBR322) as a control, because (i) linear DNA is taken up via the same mechanism irrespective of its origin (plasmid or genomic), as shown previously (Seitz and Blokesch 2013; Seitz et al. 2014); (ii) linearised plasmid DNA is expected to yield higher transformation efficiencies than genomic DNA, as it does not require chromosomal recombination and (iii) the *tetR*‐cassette used in this study to monitor BEV‐mediated HGT originates from the plasmid pBR322. The lack of transformants in the experiments containing linearised pBR322 as donor is consistent with earlier reports, demonstrating insufficient expression of the DNA uptake machinery in the absence of chitin, which acts as an activator in *V. cholerae* (Meibom et al. 2005; Lo Scrudato and Blokesch 2012; Lo Scrudato and Blokesch 2013). Together with the fact that BEVs were DNase treated before HGT, this result strongly indicates that BEV‐mediated HGT involves luminal DNA rather than external DNA.

To assess whether BEV‐mediated HGT represents a general consequence of stress‐induced vesiculation rather than being specific to bile or MMC, we examined two additional antibiotics with distinct modes of action, that is, Cm and CIP. Cm inhibits protein synthesis by reversibly binding the 50S ribosomal subunit, whereas CIP disrupts DNA replication through inhibition of DNA gyrase and topoisomerase IV. Dose–response analyses showed physiological effects comparable to bile and MMC, including reduced OD_600_ and CFU with increasing stressor concentrations under both virulence inducing (AKI) and non‐virulence inducing (LB) conditions (Figure S9a, b). As observed for bile and MMC, antibiotic exposure increased BEV‐associated protein biomass, LPS levels and nucleic acid content per µL at specific concentrations (Figure S9c–e). CIP treatment also increased BEV abundance while maintaining BEV size comparable to controls (Figure S9f–h). In contrast, Cm exposure reduced BEV numbers and significantly decreased BEV size despite increased biomass per volume, indicating distinct biophysical characteristics of Cm induced BEVs. Because nucleic acid abundance is the key determinant for BEV‐mediated HGT, we selected concentrations yielding maximal nucleic acid content and tested their transfer capacity. Using equivalent BEV quantities, both BEVs originated from antibiotic‐induced cultures resulted in vesiduction rates comparable to bile and MMC‐induced BEVs. Together, these findings indicate that antibiotic stress similarly promotes BEV mediated HGT in *V. cholerae*.

To validate the HGT results obtained with BEVs derived from VC1620/1::*tetR* with a different antibiotic resistance marker, an alternative BEV donor strain, that is, VC1620/1::*cmR*, harbouring a Cm resistance cassette in the same intergenic region between VC1620 and VC1621 was constructed. For the validation of resistance transfer mediated by BEVs we focused on bile‐induced BEVs, as bile exposure constitutes the most physiologically relevant stress condition for *V. cholerae* investigated in this study. Using bile‐induced BEVs derived from VC1620/1::*cmR* as donor material in HGT assays resulted in transformation rates comparable to those obtained with bile‐induced BEVs derived from VC1620/1::*tetR* for both recipient cultivation conditions, that is, AKI and SOC: HEPES (Figure S10a and Figure 5). In contrast, control BEVs obtained from non‐stressed VC1620/1::*cmR* cultures did not mediate BEV‐mediated HGT (Figure S10a). Thus, BEV‐mediated HGT using stress‐induced BEVs could be validated for a second resistance marker transfer, demonstrating the robustness and general applicability of BEV‐mediated HGT.

To enable comprehensive future evaluations, transformation rates were also normalised to the amount of vesicle‐associated DNA wherever applicable (Figure S10b, c). In general, analyses of DNA‐normalised transformation rates revealed trends consistent with the CFU‐based analyses: (i) higher transformation rates were observed for trypsin‐digested, bile‐induced BEVs compared to untreated bile‐induced BEVs under both recipient cultivation conditions (AKI and SOC: HEPES); (ii) comparable DNA‐normalised transformation rates were observed for bile‐ and MMC‐induced BEVs in both recipient cultivation conditions and (iii) recipient cultivation in SOC: HEPES resulted in slightly higher transformation rates than cultivation in AKI. Although some differences in transformation rates were observed when using different stress‐induced BEVs as donors, we refrain from drawing strong conclusions, as both DNA quality and concentration may vary across the different stress‐induced BEV preparations.

To assess the role of competence machinery, HGT experiments were performed with deletion mutants Δ*tfoX*, Δ*comEA* and Δ*pilA* as recipients focusing on BEV^AKI‐bile^ derived from strain VC1620/1::*tetR* as donor material (Figure 5b). TfoX represents a conserved transcriptional activator of competence genes in *Vibrionaceae* and other bacteria (Lo Scrudato and Blokesch 2013; Simpson et al. 2019; Jaskolska and Gerdes 2015). ComEA is a periplasmic protein that shuttles DNA from the outer membrane to the inner‐membrane channel ComEC (Seitz and Blokesch 2013; Chen et al. 2005). PilA is the subunit of the pseudopilus involved in DNA binding and uptake via the secretin pore PilQ (Seitz and Blokesch 2013; Lång et al. 2009). For these assays, bile‐induced BEVs were used as donor material, reflecting both their physiological relevance during the intestinal colonisation and first characterisation in this study. No detectable transformation was observed in Δ*tfoX* or Δ*comEA* under the tested cultivation conditions (i.e., AKI and SOC: HEPES), whereas the Δ*pilA* mutant exhibited a transformation rate comparable to WT (Figure 5b). This suggests that the extracellular pilus structures are dispensable, while intracellular components of the competence system are required for BEV‐mediated HGT.

Since BEV‐mediated HGT seems to be PilA‐independent, liberation of luminal DNA from the vesicles into the periplasm is a likely scenario, which requires at least a transient contact of BEVs and the recipient outer membrane. To assess BEV‐cell interaction, we adapted a fluorescence‐based uptake assay using octadecyl rhodamine B chloride (R18)‐labelled BEVs, previously established to monitor BEV internalisation in eukaryotic host cells (Zingl et al. 2021). Bile‐ and MMC‐induced BEVs, as well as BEVs isolated from control cultures without stressors, showed comparable uptake by *V. cholerae* WT (Figure 5c). Notably, trypsin‐digestion significantly increases uptake rates by approximately two‐fold, correlating with the enhanced transformation rates described above (Figure 5a). Mutants of the competence machinery, that is, Δ*comEA* and Δ*pilA*, also showed uptake rates comparable to the WT. Thus, the lack of detectable transformants in BEV‐mediated HGT assays using Δ*comEA* as recipient cannot be attributed to reduced BEV uptake dynamics.

To investigate BEV‐mediated transformation during intestinal colonisation, we adapted the adult mouse model of cholera. First, we assessed the colonisation fitness of the VC1620/1::*tetR* and the Δ*comEA* compared to the WT in competition assays. VC1620/1::*tetR* showed a slight but significant advantage during both in vitro cultivation and in vivo colonisation compared to WT (Figure 5e). We have no explanation for this general growth advantage of VC1620/1::*tetR*. In contrast, competition experiments between Δ*comEA* and WT revealed a similar fitness in vitro and in vivo. These results exclude major colonisation fitness defects in the strains for in vivo experiments that would confound BEV‐mediated transformation analyses. For the BEV‐mediated transformation assay during intestinal colonisation, mice were first infected with WT or Δ*comEA* to establish stable colonisation. Subsequently, bile‐induced BEVs derived from VC1620/1::*tetR* were administered by four oral doses over two days. On Day 4, mice were euthanised and intestinal contents were analysed for transformation events. Robust colonisation was observed in all animals ranging from 1.18 to 5.14 × 10^5^ total CFU (SmR) per mouse, excluding insufficient colonisation as a limiting factor. Using this protocol, transformation rates significantly above the detection limit were observed for all mice colonised with *V. cholerae* WT as recipient (Figure 5f). Consistent with the in vitro HGT assay, no transformation was detected in mice colonised with Δ*comEA* as recipient. Thus, during in vivo colonisation of *V. cholerae* bile‐induced BEVs can serve as DNA donors for intestinal HGT, which requires at least parts of the competence machinery.

## 4 Discussion

The biogenesis of Gram‐negative BEVs via blebbing from the outer membrane seems a general phenomenon in non‐stressed bacterial cultures (Toyofuku et al. 2019; Zingl et al. 2020; Kulp and Kuehn 2010). Although blebbing‐type BEVs are enriched by outer membrane and periplasmic components, several studies on Gram‐negative BEVs have also reported the presence of cytoplasmic proteins and nucleic acid in their cargo (Langlete et al. 2019; Pérez‐Cruz et al. 2013, 2015). This was generally attributed to cell death and lysis of a small sub‐population, which can be enhanced by exposure to stressors, such as genotoxins, antibiotics, extreme temperatures or UV light (Turnbull et al. 2016; Fulsundar et al. 2014; Li et al. 2024; Bauwens et al. 2017; Heredia‐Ponce et al. 2023). In this study, we demonstrate that the facultative human pathogen *V. cholerae* releases such explosive‐type BEVs upon exposure to antimicrobial stressors under both virulence and non‐virulence inducing conditions. Formation of explosive‐type BEVs was triggered not only by the prototypical genotoxic agent MMC, the protein biosynthesis inhibitor Cm, the DNA replication inhibitor CIP but also by bile, a host‐derived antimicrobial compound present in the mammalian intestinal tract. Notably, bile is a well‐established in vivo trigger of the *V. cholerae* virulence cascade and the concentrations used in this study are within the physiological range reported for the human intestine (Bergström et al. 2014; Cho et al. 2021). Thus, bile likely represents a natural trigger for explosive‐type BEV formation during *V. cholerae* colonisation of the human gut.

Compared to control BEVs, bile‐ and MMC‐induced BEVs were larger and contained substantial amounts of cytoplasmic proteins and nucleic acids. Despite these similarities, the two types of stress‐induced BEVs also displayed notable differences regarding their composition and biogenesis. Proteomic analyses revealed distinct protein profiles for bile‐ versus MMC‐induced BEVs. MMC induced the SOS response in *V. cholerae*, which is known to trigger prophage activation, leading to cell lysis, explosive‐type BEV formation and phage release (Baharoglu and Mazel 2011; Otsuji et al. 1959; Mohanraj and Mandal 2022; Wagner et al. 2002; Owen et al. 2017). Consistent with this, proteomic analyses identified phage‐associated proteins such as RstB1/2 or RstA exclusively in MMC‐induced BEVs, and TEM analyses revealed phage‐like structures only in MMC‐derived BEV preparations. Thus, explosive‐type BEVs formed upon MMC exposure in *V. cholerae* likely arise from DNA damage‐induced prophage activation, as reported for other bacterial species (Toyofuku et al. 2019; Toyofuku et al. 2023; Turnbull et al. 2016). Although bile can cause some degree of DNA damage in *V. cholerae* (Wessel et al. 2025), bile exposure did not activate the SOS response, and TEM analyses of bile‐induced BEV preparations revealed no evidence of phage particles. Instead, compared to bile‐induced BEVs, MMC‐induced BEVs displayed greater protein complexity, with higher numbers of proteins identified, particularly those localised in the cytoplasmic membrane and cytoplasm. Given bile's emulsifying properties, we speculate that bile‐triggered BEV formation results from membrane destabilisation and subsequent lysis rather than prophage activation. In aqueous solutions, bile salts can self‐assemble into micelles ranging in diameters from 6.2 to 20 nm, depending on various parameters, such as bile composition, concentration and pH (Reuben et al. 1982; Borgstrom 1965). Although these diameters fall within the detection range of our size measurements, no particles with this size range were detected in the BEV preparations used in this study. Therefore, it is likely that bile components are incorporated into BEVs during BEV biogenesis. Thus, explosive‐type BEVs may emerge through distinct mechanisms, giving rise to different BEV subtypes.

The detection of nucleic acids in stress‐induced BEVs suggests a potential role in HGT in *V. cholerae*. Several recent studies have reported BEV‐mediated HGT in Gram‐negative bacteria, including *E. coli*, *Acinetobacter baumannii*, *Thermus thermophilus*, *Pseudomonas aeruginosa* and *Klebsiella pneumoniae* (Dell'Annunziata et al. 2021; Marinacci et al. 2023). Although these studies provide compelling evidence for BEV‐mediated transfer of plasmids, evidence for the transfer and stable maintenance of chromosomal genetic elements remains scarce and often difficult to interpret. For example, BEV‐mediated HGT of chromosomally encoded virulence factors was reported for *E. coli* O157:H7, but the functional expression of the transferred effectors was not tested (Kolling and Matthews 1999). Moreover, phage‐mediated transduction events cannot be entirely excluded, as some of the investigated genes, that is, *stx1* and *stx2*, are phage‐encoded. Another study highlights the transfer of several chromosomal antibiotic resistance genes in *Avibacterium paragallinarum*, but the genetic exchange was only transient, as resistance was lost after one passage (Xu et al. 2022). In contrast, in our BEV‐mediated HGT assays, we deliberately inserted a TetR resistance gene into an intergenic chromosomal region that has no obvious association to mobile elements such as phage sequences or insertion elements. Our results not only demonstrate reproducible, PCR‐validated transmission of a non‐mobile chromosomal antibiotic resistance cassette via stress‐induced BEVs but also confirm the functional expression of the acquired gene, which was stably maintained across multiple passages. To our knowledge, persistent BEV‐mediated HGT of chromosomal sequences with subsequent functional gene expression by the recipient was so far only reported for *Porphyromonas gingivalis*, using an erythromycin‐resistance cassette inserted in the chromosomally encoded *fimA* gene (Ho et al. 2015).

To gain deeper mechanistic insights of BEV‐mediated HGT, we analysed the influence of several parameters, such as BEV dosage, recipient inoculum density and growth conditions. Overall, our results indicate that lower recipient starting densities and nutrient‐rich growth medium enhance BEV‐mediated HGT rates. However, the parameters appear to interact, underscoring the need to evaluate them in combination. Transformation by uptake of free DNA present in the BEV preparations is unlikely, as BEVs were DNase‐treated before use and linearised plasmids as a control condition failed to produce detectable transformants. Likewise, control BEVs purified from unstressed *V. cholerae* cultures contained negligible amounts of nucleic acids and did not reproducibly mediate BEV‐mediated HGT, reinforcing the importance of stress‐induced explosive‐type BEVs. Substantial impact of nanoparticles such as phages, which were observed in MMC‐induced BEV preparations, is rather unlikely as bile‐induced BEVs exhibit a comparable HGT frequency without any detection of phage‐like structures in the TEM or prophage‐associated proteins in the LC‐MS/MS analyses. Importantly, successful BEV‐mediated HGT was not only achieved in vitro, but also in the mouse intestine at relatively high rates. This may reflect a ‘snowball effect’: Once initial HGT events occur, intestinal conditions such as constant bile exposure may promote formation of new stress‐induced BEVs carrying the *tetR*‐cassette, enabling subsequent rounds of transfer. Notably, the highest uptake and HGT rates in vitro were achieved using trypsin‐digested BEVs. It should be emphasised that BEVs were routinely subjected to heat treatment during preparation for HGT assays to ensure inactivation of DNases. This procedure likely induces irreversible protein denaturation, resulting in the loss of native tertiary structures. In contrast, treatment with trypsin leads to the proteolytic cleavage of accessible protein domains, thereby disrupting primary protein structures, particularly those exposed on the vesicle surface. Consequently, trypsin digestion is expected to alter key biophysical properties of BEVs, including surface charge distribution and membrane accessibility. Importantly, trypsin digestion represents a physiologically relevant condition, as BEVs in the human gastrointestinal tract are naturally exposed to proteolytic enzymes. Overall, these findings indicate that surface‐associated proteins may hinder BEV‐recipient interactions and further suggest that intestinal proteolytic conditions can enhance BEV‐mediated HGT in vivo.

**FIGURE 6:**
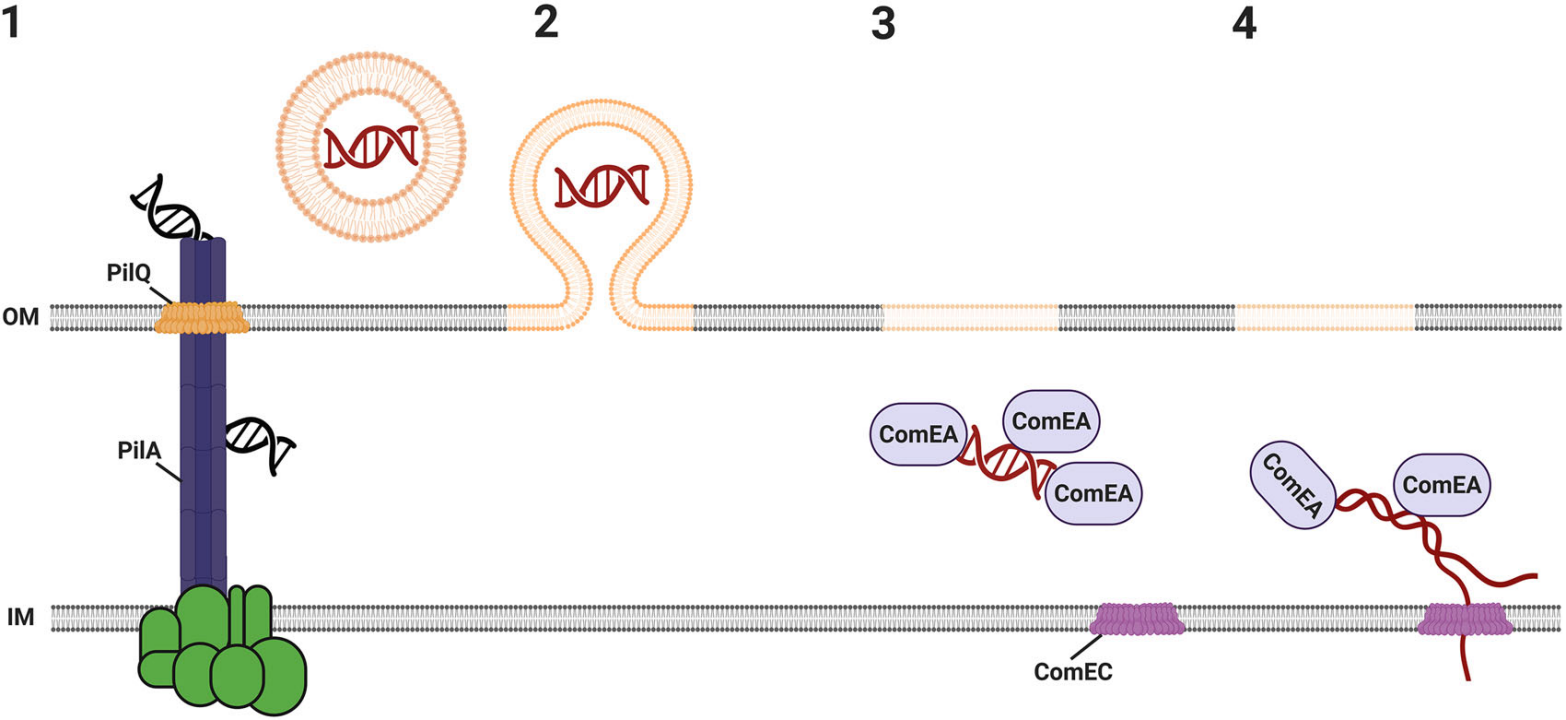
Proposed model for BEV‐mediated HGT in *V. cholerae*. Stress‐induced BEVs liberate their luminal DNA content into the periplasm. The vesicle content is most likely released by (transient) fusion with the outer membrane of the recipient cell (Deatherage and Cookson 2012). Thus, the BEV‐mediated DNA transfer does not require the exterior parts relevant for DNA import via the outer membrane (Gill et al. 2019). This includes the surface‐exposed pili with PilA being the major pilin subunit. Periplasmic ComEA directs the DNA to the downstream DNA‐uptake machinery including the inner membrane channel ComEC resulting in transport to the cytosol (Toyofuku et al. 2019, 2023). The internalised DNA can integrate into the chromosome via RecA‐dependent homologous recombination. Created in BioRender. Fleischhacker (2025) https://BioRender.com/xve8fcp. BEV, bacterial extracellular vesicle; HGT, horizontal gene transfer.

Finally, assays using defined mutants provided further insights and allowed us to propose a first mechanistic model (Figure 6). Extracellular components of the DNA uptake machinery, such as PilA, proved dispensable, whereas the periplasmic ComEA complex and downstream elements were essential (Seitz et al. 2014; Seitz and Blokesch 2014). These results further support the conclusion that the herein observed HGT is BEV‐mediated rather than phage‐mediated, although this cannot be definitely confirmed. For phage‐mediated HGT to play a relevant role, the periplasmic located protein ComEA would need to function as a phage receptor, an interaction for which no evidence currently exists. Together with the uptake assays, these findings rather support a model in which BEVs transiently fuse with the recipient, which is sufficient for DNA release into the periplasm. The correlation between BEV internalisation and HGT rates, especially with trypsin‐digested BEVs, further supports this scenario. From there, ComEA and ComEC direct the DNA to the inner membrane for cytosolic import. Stable maintenance requires chromosomal integration, most likely via RecA‐dependent homologous recombination. This model aligns with prior reports on BEV‐mediated plasmid transfer in *Acinetobacter baylyi*, which also requires a functional competence system (Fulsundar et al. 2014). Moreover, a recent study on the Gram‐positive bacterium *Streptococcus pneumoniae* demonstrated that BEV‐mediated HGT depends on the transformation machinery (Smith et al. 2024), suggesting conserved mechanistic principles of ‘vesiduction’ across Gram‐negative and Gram‐positive bacteria.

In summary, this study identifies stress‐induced BEVs as potent vehicles for intra‐species HGT of chromosomal sequences both in vitro and in vivo. Beyond the classic genotoxic agent MMC as well as the antibiotics Cm and CIP we established bile as a host‐derived trigger of explosive‐type BEV formation in *V. cholerae*, suggesting that such BEVs arise naturally during intestinal colonisation. The proteolytic environment and host‐derived stressors, such as bile, further enhance BEV‐mediated HGT. The evolution of *V. cholerae* from a benign marine bacterium into a virulent human pathogen is well recognised as being driven by successive HGT events (Reidl and Klose 2002). Our findings support the idea that BEV‐mediated HGT contributes to this evolutionary process and may facilitate the dissemination of virulence and antibiotic resistance genes in *V. cholerae* and other intestinal pathogens.

## Author Contributions

**Dominik Fleischhacker**: investigation, formal analysis, resources, visualisation, writing – original draft, writing – review and editing. **Yi‐Chi Chen**: investigation, formal analysis. **Noa Sanchez Gordo**: investigation, formal analysis. **Amar Cosic**: formal analysis. **Ratchara Kalwong**: investigation. **Leo Eberl**: supervision, writing – review and editing. **Stefan Schild**: conceptualisation, funding acquisition, supervision, writing – original draft, writing – review and editing.

## Funding

This research was funded in whole, or in part, by the Austrian Science Fund (FWF) grants [10.55776: P33073 and 32577] to S.S. For the purpose of open access, the author has applied a CC BY public copyright licence to any Author Accepted Manuscript version arising from this submission. The authors acknowledge the financial support by the University of Graz.

## Conflicts of Interest

The authors declare no conflicts of interest.

## Supporting information




**Supporting Information**: jev270301‐sup‐0001‐SuppMat.docx

**Supporting Information**: jev270301‐sup‐0002‐tableS3.xlsx

**Supporting Information**: jev270301‐sup‐0003‐tableS5.xlsx

## Data Availability

The data that support the findings of this study are available from the corresponding author upon reasonable request.
