# Supplementary material for: Host Factor Induced Bacterial Extracellular Vesicles Promote Horizontal Gene Transfer in Vibrio cholerae
Source: J Extracell Vesicles. 2026 May 9;15(5):e70301. doi: 10.1002/jev2.70301 (PMC13157587; doi:10.1002/jev2.70301)
Supplement: Supplementary file 1 — Supporting Information: jev270301‐sup‐0001‐SuppMat.docx [file JEV2-15-e70301-s001.docx]

**Supporting Information**

**Table S1. Strains and plasmids used in this study.**

| **Bacterial strain** | **Description** | **Reference** |
| --- | --- | --- |
| *E. coli* |  |  |
| DH5αλpir | *Escherichia coli*, F^-^ *endA1* *glnV44 thi-1 recA1 relA1 gyrA96 deoR nupG* Φ80∆*lacZ*ΔM15 Δ(*lacZYA*-*argF*) U169 *hsdR17*(_rK_ ^-^ _mK_^+^) λpirRK6 | (1) |
| Sm10λpir | *E. coli,* *thi thr leu tonA lacY supE recA*::RPA-2-Te::Mu λpir, KmR | (1) |
| *V. cholerae* |  |  |
| WT | *Vibrio cholerae* spontaneous streptomycin resistant (SmR) mutant of E7946 (O1 El Tor Inaba, clinical isolate from Bahrain),hapR+ SmR | (2) |
| *lacZ^-^* | insertion of res1-neo-sacB-res1 in *lacZ* of WT | (3) |
| VC1620/1::*tetR* | insertion of a tetracycline-resistance gene originating from pBR322 between the genes VC1620/1 in WT | This study |
| ∆*tfoX* | deletion of VC1153 in WT | This study |
| ∆*comEA* | deletion of VC1917 in WT | This study |
| ∆*pilA* | deletion of VC2423 in WT | This study |
| **Plasmids** | | |
| precN-gfp | green fluorescent protein gene (*gfp*) fused to the *recN* promoter, ApR | (4) |
| pCVD442 | *ori6K*, *mobRP4*, *sacB*, ApR | (5) |
| pVC1620/1::*tetR* | pCVD442 with up- and downstream fragments of the end and intergenic region of VC1620 flanking the tetracycline resistance gene originating from pBR322 | This study |
| pVC1620/1::*cmR* | pCVD442 with up- and downstream fragments of the end and intergenic region of VC1620 flanking the tetracycline resistance gene originating from pBAD33 | This study |
| p∆comEA | pCVD442 with up- and downstream fragments of *comEA*, ApR | This study |
| p∆pilA | pCVD442 with up- and downstream fragments of *pilA*, ApR | This study |
| pBR322 | wide host range, low copy number, ColE1-type plasmid, ApR, TetR | (6) |
| pBAD33 | arabinose-inducible plasmid, low copy number, p15A origin, CmR | (7) |

**Table S2. Oligonucleotides used in this study.**

| VC1620/1_SacI_1 | TATGAGCTCTCAATGCTTATTATGAAGGCAC |
| --- | --- |
| VC1620/1_KpnI_2 | TATGGTACCGGGATTTTTATTAAAGGCAAGTG |
| TetR_KpnI | AAAGGTACCTTCCATTCAGGTCGAGGT |
| TetR_EcoRI | AAAGAATTCATGTTTGACAGCTTATCATCG |
| VC1620/1_EcoRI_3 | TTTGAATTCTAACTGGCGGGATTTTTGTC |
| VC1620/1_XbaI_4 | TATTCTAGAGGTTGGGAAATGGTCACACTC |
| TfoX_XbaI_1 | ATATCTAGACAACTGTTCAATAGCTTGCGC |
| TfoX_EcoRI_2 | ATAGAATTCGTTCCCCGATTAAGGAAGATCAC |
| TfoX_EcoRI_3 | TTTGAATTCGTTAAAGCGTTAGCCACGTTC |
| TfoX_SacI_4 | TTTGAGCTCGAACCCGGAGTCGATTTTAAG |
| ComEA_XbaI_1 | AAATCTAGACCCTTGATAGTTTCTATGCTAAGC |
| ComEA_EcoRI_2 | TTTGAATTCGATAGACCCTCATTTTGGTTGTTG |
| ComEA_EcoRI_3 | ATAGAATTCGCTGCAATCATGTGTCTCG |
| ComEA_SacI_4 | ATAGAGCTCGCGGTCGTCTCTTTCCTG |
| PilA_SacI_1 | AAAAGAGCTCAAAGGCACCC |
| PilA_EcoRI_2 | TTTGAATTCATGCCTTGCTACACAA |
| PilA_EcoRI_3 | AAAGAATTCTGCTCACCAACCTTGTTGC |
| PilA_XbaI_4 | AAATCTAGAGACCAAGATGCCATCGCAG |
| VC1620/1_BamH1_3 | TTTGGATCCTAACTGGCGGGATTTTTGTC |
| CmR_KpnI | TTTGGTACCTTACGCCCCGCCCTGCC |
| CmR_BamHI | AAATGGATCCTGATCGGCACGTAAGAGG |

* restriction sites are underlined

**Table S4. Comparative enrichment analysis of SOS regulons identified by mass spectrometry (MS) of whole cell lysates (WCL) derived from bile- or MMC-exposed cultures compared to non-stressed control conditions.**

WCL were obtained from *V. cholerae* WT cultivated in virulence (AKI) and non-virulence inducing conditions (LB) presence of bile (17.25 mM) or MMC (60 ng ml^-1^) or without any stressor (control, co) resulting in the sample sets: AKI^bile^, AKI^MMC^, AKI^co^, LB^bile^, LB^MMC^ and LB^co^. Shown are the individual ratios of the mean “score” values for selected SOS-regulon associated proteins obtained by LC-MS/MS analysis of three biological replicates (n=3). Proteins were included if detected in ≥2 of 3 replicates for the respective condition. 2-fold enrichment of proteins is highlighted in bold.

| **VC number^1^** | **protein annotation^1^** | **AKI^bile^ /**  **AKI^co^** | **AKI^MMC^ /**  **AKI^co^** | **LB^bile^ /**  **LB^co^** | **LB^MMC^ /**  **LB^co^** |
| --- | --- | --- | --- | --- | --- |
| VC0082 | DNA recombination protein RmuC | 1.0525 | **6.0451** | 0.7944 | **9.5456** |
| VC0083 | Ubiquinone/menaquinone biosynthesis methyltransferase UbiE | 0.5460 | 1.2917 | 0.9093 | 1.2651 |
| VC0085 | ubiquinone biosynthesis protein UbiB | 1.3425 | 1.2748 | 1.4614 | **3.7049** |
| VC0086 | Sec-independent protein translocase TatA | **2.5708** | **3.2761** | **2.5168** | **3.8709** |
| VC0087 | Sec-independent protein translocase protein TatB | 0.0023 | 0.5274 | 0.9170 | 0.6073 |
| VC0092 | lexA repressor | **2.0815** | 0.3099 | 0.2420 | 0.1596 |
| VC0190 | DNA helicase II | 1.9514 | **3.2628** | 1.9015 | **3.5655** |
| VC0394 | UvrABC system protein A | 0.6442 | **2.8028** | 1.4609 | **5.9245** |
| VC0397 | Single-stranded DNA-binding protein | 0.3259 | 0.7757 | 0.4835 | 0.4391 |
| VC0543 | Protein recA | 1.8482 | **7.0485** | 1.3485 | **4.7585** |
| VC0851 | small protein A | **3.9468** | **4.1338** | 1.6145 | 0.9708 |
| VC0852 | DNA repair protein recN | 1.6258 | **7.0260** | 0.5793 | **7.3947** |
| VC1190 | putative phosphoribosylaminoimidazole-succinocarboxamide synthase PurC | 0.4147 | 1.5557 | 1.0538 | 0.7944 |
| VC1191 | putative Superfamily II DNA and RNA helicase | 1.4023 | 1.0301 | 0.2550 | **33.9596** |
| VC1370 | putative Signal transduction histidine kinase domain, Methyl-accepting chemotaxis domain and GGDEF family protein | **2.5462** | 1.6091 | 0.3000 | **2.6982** |
| VC1453 | RstB phage-related integrase | 0.1900 | **5.4526** | **2.4657** | 1.5195 |
| VC1454 | RstA phage-related replication protein | 1.6319 | **3.3375** | 0.8098 | **3.3530** |
| VC1455 | Cryptic phage CTXphi transcriptional repressor rstR | 0.2785 | 0.2881 | 0.4581 | 0.2036 |
| VC1845 | Holliday junction ATP-dependent DNA helicase ruvB | 1.9543 | **3.0014** | 1.5607 | **5.5040** |
| VC2287 | DNA polymerase IV | 0.7290 | **2.0607** | 1.2932 | **9.5792** |
| VC2326 | dsDNA-binding SOS-regulon protein | 0.0591 | 1.6190 | 1.8829 | 0.0286 |
| VC2711 | ATP-dependent DNA helicase recG | 0.2200 | 0.2994 | 1.0841 | **4.8929** |
| VCA0291 | Site-specific recombinase IntIA | **2.3369** | **7.0240** | 1.2662 | **8.3295** |

^1^ according to KEGG (8)


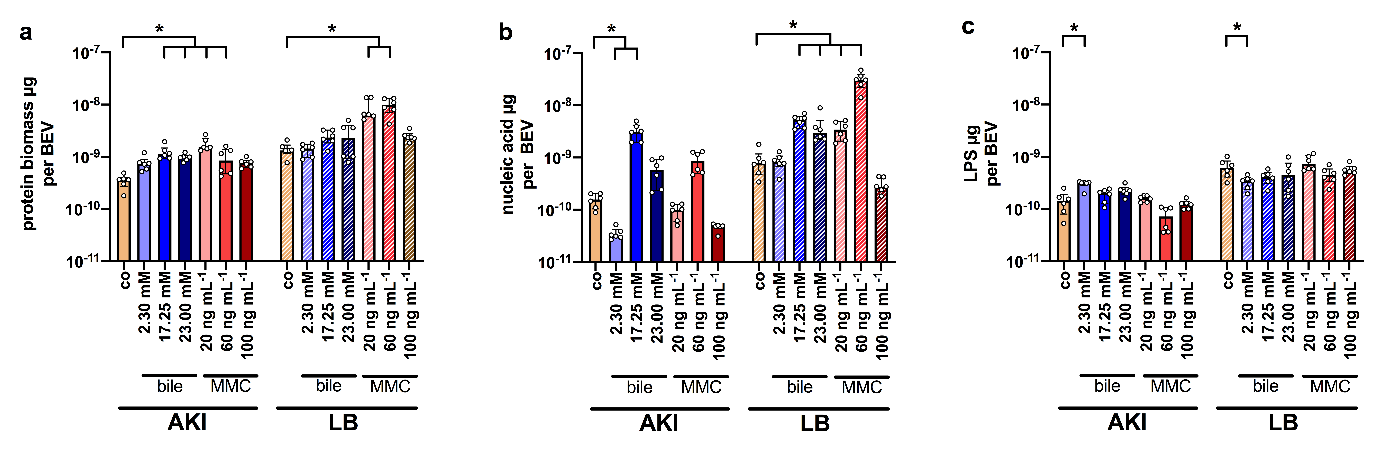


**Supplementary Figure S1: Assessment of additional content parameters of the BEV derived from control and stressor-exposed *V. cholerae* cultures.**

**a,** Total protein biomass of the BEV preparations normalized to the BEV amount determined by nanoparticle tracking analysis (NTA).

**b,** Nucleic acid amount of the BEV preparations normalized to the BEV amount determined by nanoparticle tracking analysis (NTA).

**c,** LPS amount of the BEV preparations normalized to the BEV amount determined by nanoparticle tracking analysis (NTA).

**a-c,** Data is presented as median ± interquartile range (IQR). For the LB and AKI data sets significant differences between the control (co) and stress-induced samples were analyzed by Kruskal–Wallis with uncorrected Dunn´s multiple comparison test (*, *P* < 0.05, n=6).


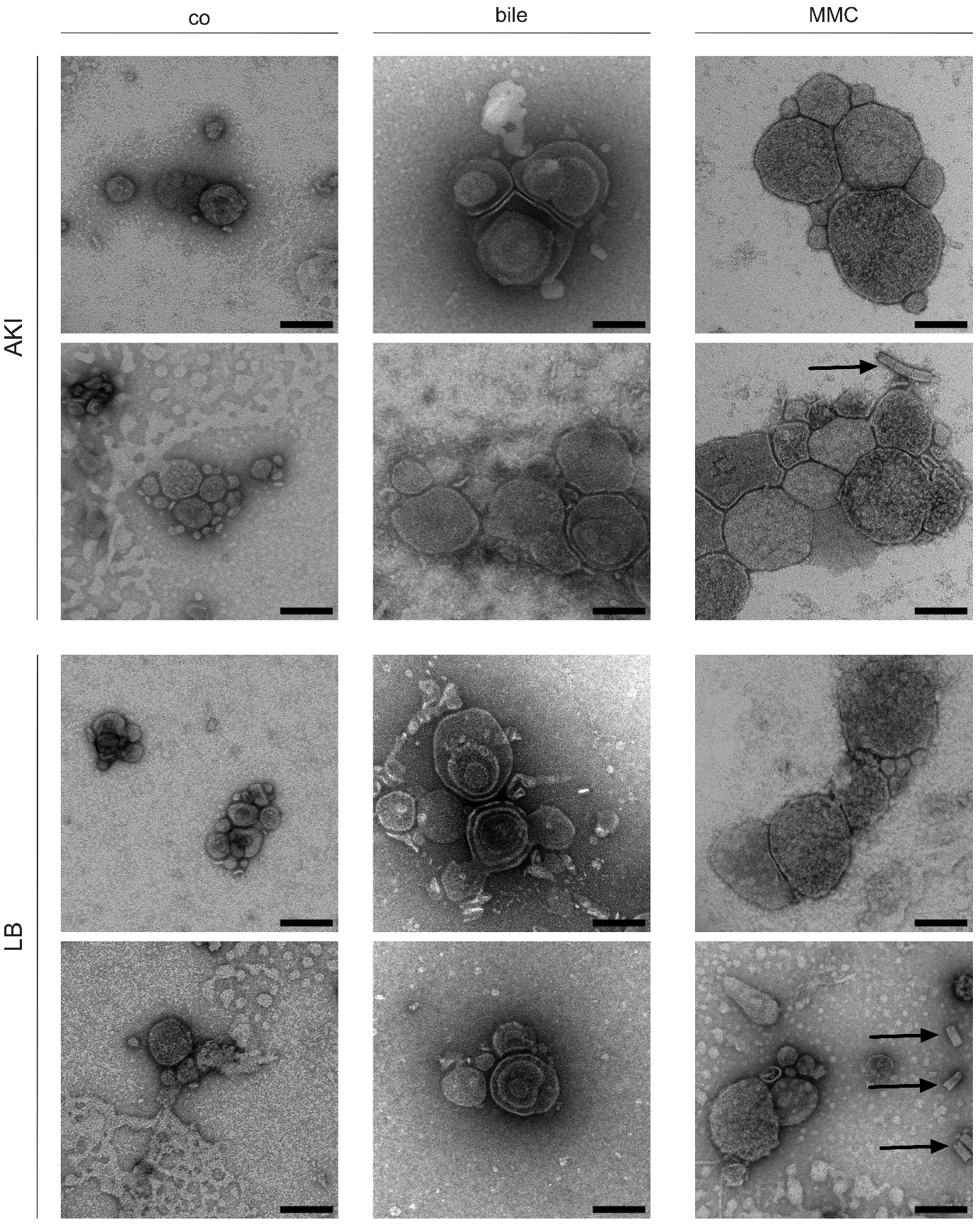


**Supplementary Figure S2: Visualization of the BEVs derived from control and stressor-exposed *V. cholerae* cultures.**

Shown are representative transmission electron microscopy (TEM) images visualizing the different BEVs used in this study (two images per condition). BEVs were obtained from *V. cholerae* WT cultivated in virulence (AKI) and non-virulence (LB) inducing conditions presence of bile (17.25 mM) or MMC (60 ng ml^-1^) or without any stressor (control, co). The presence of phages is highlighted with black arrows. The scale bars represent 100 nm.


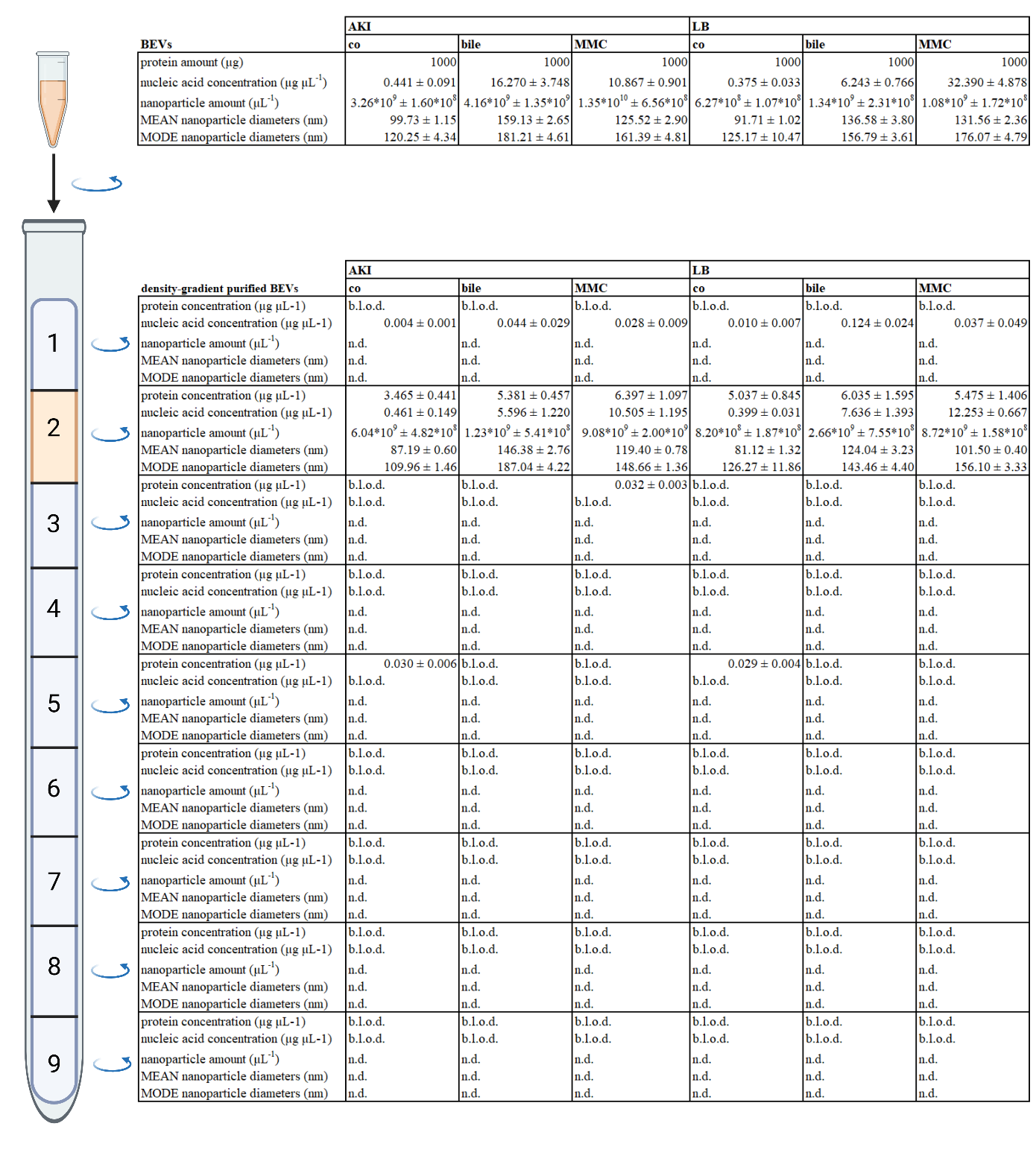


**Supplemental Figure S3: Evaluation of density gradient purification.**

BEVs were obtained from *V. cholerae* WT cultivated under virulence (AKI) and non-virulence inducing condition (LB) in presence of bile (17.25 mM) or MMC (60 ng mL^-1^) or without any stressors (control, co) and analyzed for protein concentration (Bradford), nucleic acid concentration (SYTO-9), particle amount (NTA) as well as the mean and mode particle diameters (ZetaSizer). Original BEV preparations were subjected on a density-gradient and separated into 9 discrete fractions (for details see “Material and Methods: Density gradient purification of BEVs”). Each fraction was quantified for protein concentration (Bradford), nucleic acid concentration (SYTO-9), with only fraction 2 containing substantial amounts of proteins and nucleic acids. Thus, nanoparticle amount and diameters (given as mean and mode) were further assessed for fraction 2, but not for all other fractions (not determined = n.d.). Data is presented as mean ± SD (n=3). “b.l.o.d.” (limit of detection) refers to datasets were two or more datasets were below limit of detection (0.02 µg µL^-1^ for protein and 0.0006875 µg µL^-1^ for nucleic acid concentration). Created in BioRender. Fleischhacker, D. (2025). <https://BioRender.com/qjjoc4b>


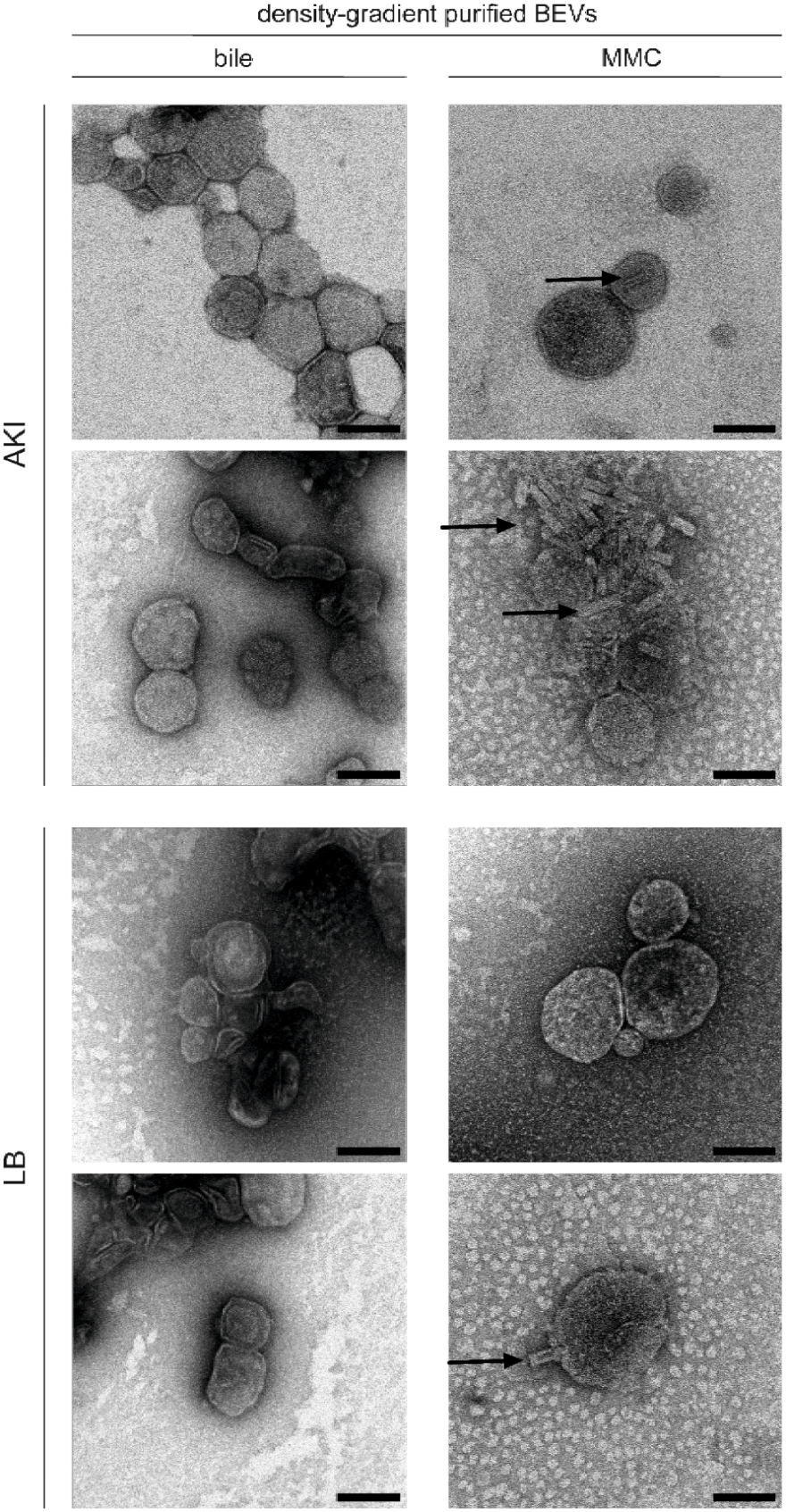


**Supplementary Figure S4: Visualization of density-gradient purified BEVs derived from stress-exposed *V. cholerae* cultures.**

Shown are representative transmission electron microscopy (TEM) images visualizing the density-gradient purified stress-exposed BEVs used in this study (two images per condition). BEVs were obtained from *V. cholerae* WT cultivated in virulence (AKI) and non-virulence inducing conditions (LB) presence of bile (17.25 mM) or MMC (60 ng ml^-1^). The presence of phages is highlighted with black arrows. The scale bars represent 100 nm.


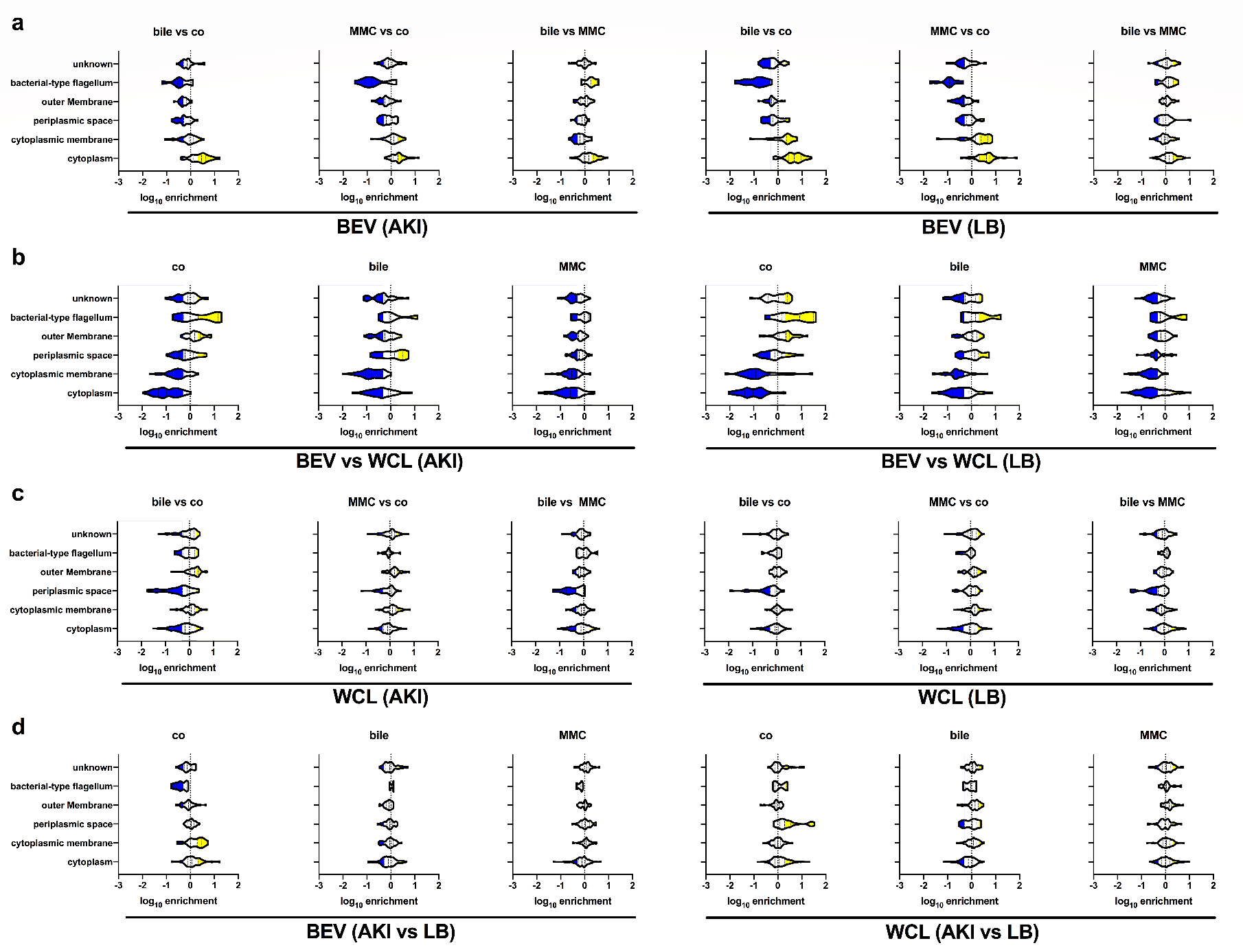


**Supplementary Figure S5: Comparative enrichment analysis of proteins identified by mass spectrometry (MS).**

**a,** Violin plots displaying the log_10_ enrichment of proteins in the BEV preparations derived from either AKI or LB conditions relative to the corresponding BEV preparations grown with presence of a different stressor (bile or MMC). Proteins are categorized by their cellular localization. BEVs were obtained from *V. cholerae* WT cultivated in virulence (AKI) and non-virulence inducing conditions (LB) presence of bile (17.25 mM) or MMC (60 ng ml^-1^) or without any stressor (control, co). 2-fold enrichment or reduction of proteins in the BEV preparations are highlighted by yellow and blue, respectively.

**b,** Violin plots displaying the log_10_ enrichment of proteins in BEV preparations relative to the corresponding WCL categorized by their cellular localization. BEVs and WCL were obtained from *V. cholerae* WT cultivated in virulence (AKI) and non-virulence inducing conditions (LB) presence of bile (17.25 mM) or MMC (60 ng ml^-1^) or without any stressor (control, co). 2-fold enrichment or reduction of proteins in the BEV preparations are highlighted by yellow and blue, respectively.

**c,** Violin plots displaying the log_10_ enrichment of proteins in WCL derived from either AKI or LB conditions relative to the corresponding WCL preparations grown in presence of a different stressor (bile or MMC). Proteins are categorized by their cellular localization. WCL were obtained from *V. cholerae* WT cultivated in virulence (AKI) and non-virulence inducing conditions (LB) presence of bile (17.25 mM) or MMC (60 ng ml^-1^) or without any stressor (control, co). 2-fold enrichment or reduction of proteins in the BEV preparations are highlighted by yellow and blue, respectively.

**d,** Violin plots displaying the log_10_ enrichment of proteins in samples (BEV preparations or WCL) derived from virulence (AKI) cultivation relative to the corresponding samples derived from non-virulence inducing (LB) cultivation. Proteins are categorized by their cellular localization. BEVs and WCL were obtained from *V. cholerae* WT cultivated in virulence (AKI) and non-virulence inducing conditions (LB) presence of bile (17.25 mM) or MMC (60 ng ml^-1^) or without any stressor (control, co). 2-fold enrichment or reduction of proteins in the BEV preparations are highlighted by yellow and blue, respectively.

**a-d,** LC-MS/MS analysis of three biological replicates (n=3). Proteins were included if detected in ≥2 of 3 replicates for the respective condition.


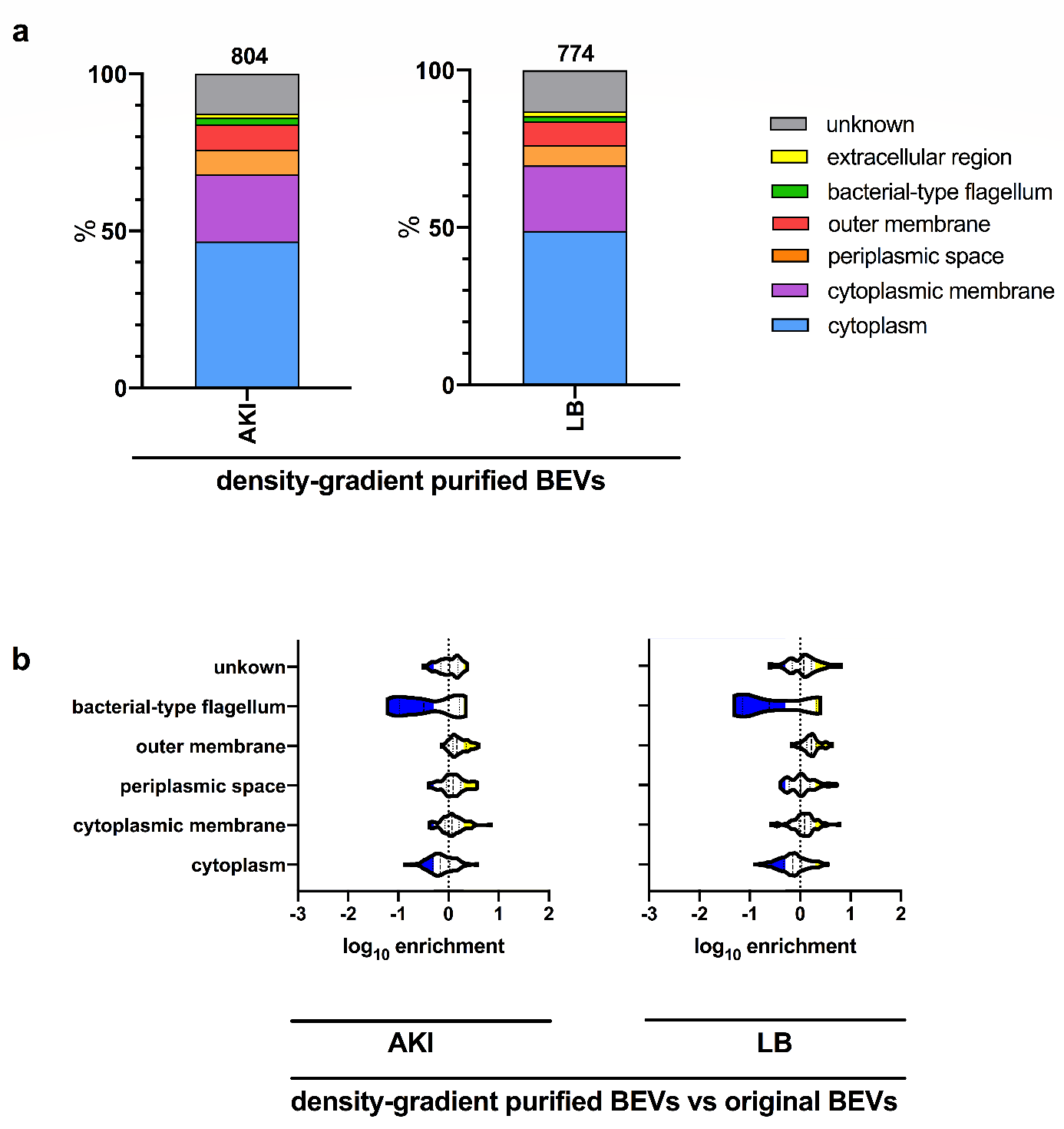


**Supplementary Figure S6: Proteomic analysis of density-gradient purified BEVs derived from bile-exposed *V. cholerae* cultures.**

**a,** Stacked bar charts displaying the percentage distribution of the predicted cellular localization for the proteins identified in the density-gradient purified BEV obtained from *V. cholerae* WT cultivated in virulence inducing (AKI) and non-virulence inducing (LB) condition in the presence of bile (17.25 mM). The total number of identified proteins is given at the top of each bar.

**b,** Violin plots displaying the log_10_ enrichment of proteins in the density-gradient purified BEVs fraction compared to the original BEVs fraction derived from *V. cholerae* WT cultivated in virulence inducing (AKI) and non-virulence inducing (LB) conditions in the presence of bile (17.25 mM). Proteins are categorized by their cellular localization. 2-fold enrichment or reduction of proteins in the BEV preparations are highlighted by yellow and blue, respectively.

**a and b,** LC-MS/MS analysis of three biological replicates (n=3). Proteins were included if detected in ≥2 of 3 replicates for the respective condition.


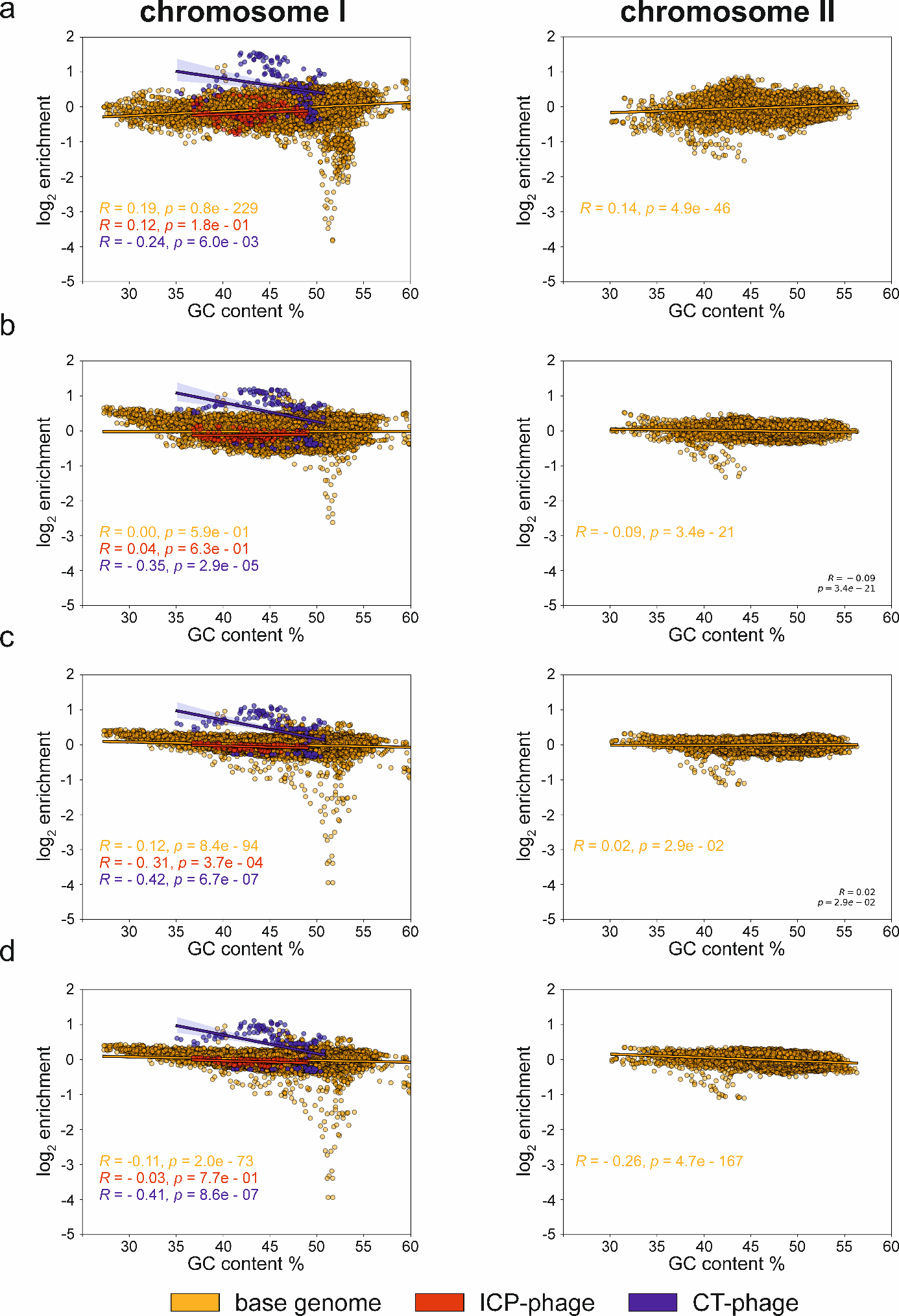


**Supplementary Figure S7: Log_2_ DNA enrichment of kbp partitions of the *V. cholerae* genome and GC-content.**

Shown are the relative abundancies of chromosomal regions in the luminal DNA of BEVs with their corresponding GC-content for chromosome 1 (left) and chromosome 2 (right). BEVs were isolated from *V. cholerae* WT cultivated in virulence (AKI) and non-virulence inducing conditions (LB) presence of bile (17.25 mM) or MMC (60 ng mL^-1^). The correlation of GC-content with the corresponding log_2_ fold change for different genomic section was calculated via Pearson correlation coefficients for each sections. Blue color denotes sections within the CT-phage, red with the ICP-phage and orange denotes the remaining genome. For each condition, BEVs derived from 6-8 independent biological replicates were pooled prior to DNA extraction and sequencing to ensure sufficient material for downstream analysis (resulting in the sequencing analysis of n=1 per condition).


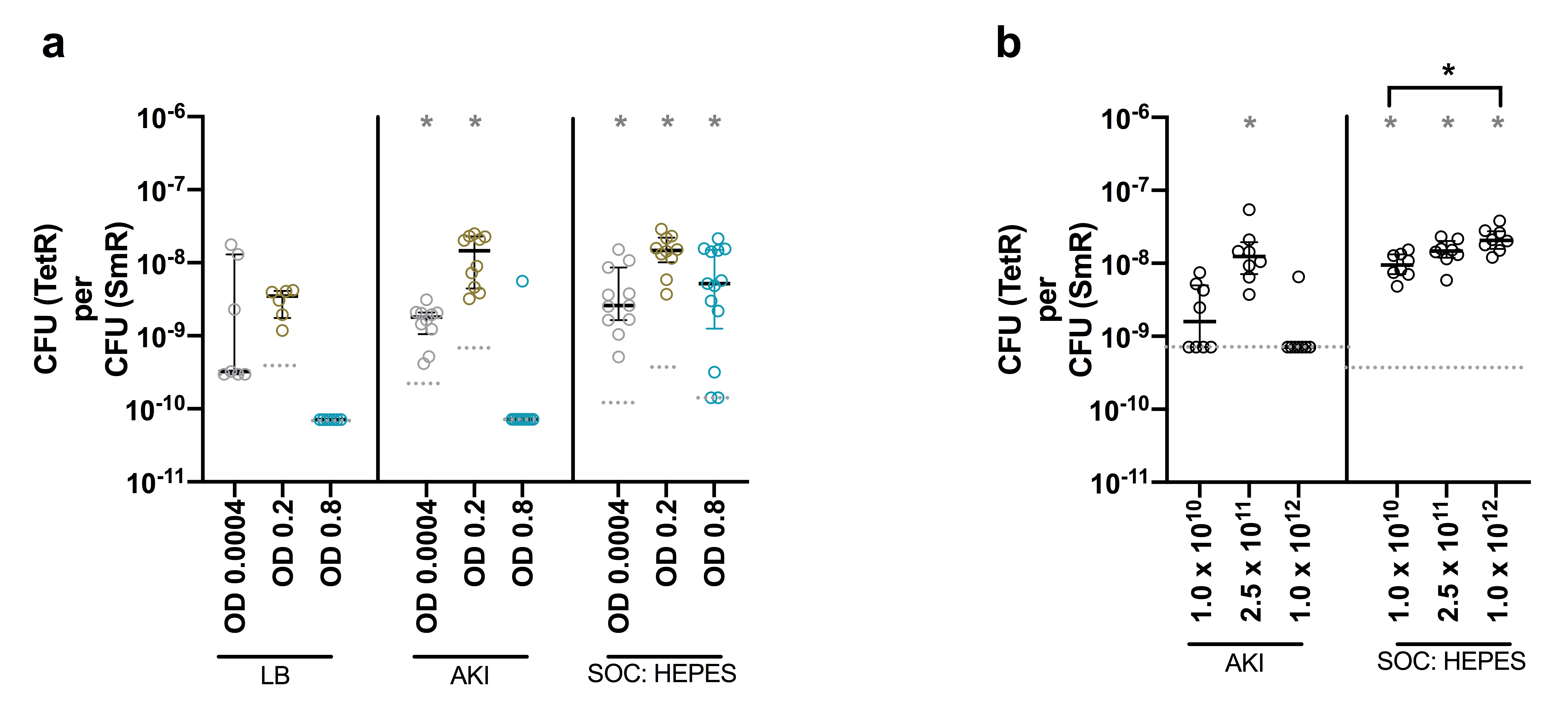


**Supplementary Figure S8: Impact of recipient cultivation, starting OD_600_ and BEV amount on BEV-mediated HGT rates.**

HGT rates of the *tetR*-cassette using BEVs^VC1620/1::^*^tetR^* derived from AKI cultures in the presence of bile (17.25 mM) as donor and *V. cholerae* WT as recipient.

**a,** Differential growth condition (LB, AKI or SOC: HEPES) and starting OD_600_ (0.004, 0.2 or 0.8) of the recipient are indicated. The recipient were incubated for 20 h at 30°C and 150 rpm with respective BEVs^VC1620/1::^*^tetR^* (2.5 x 10^11^ particles) before total CFU and transformants were determined by plating on LB-Sm and LB-Tet plates.

**b,** The recipient was grown in AKI (left panel) or SOC: HEPES (right panel), diluted to a starting OD_600_ of 0.2 and incubated with the differential amounts of BEVs^VC1620/1::^*^tetR^* (1 x 10^10^, 2.5 x 10^11^, 1 x 10^12^ particles) as indicated, before total CFU and transformants were determined by plating on LB-Sm and LB-Tet plates.

**a and b,** Data is presented as median ± interquartile range. Assays yielding in no transformants on LB-Tet were set to limit of detection (LOD), which is indicated by a dotted line. The limit of detection (LOD) was defined as 0.5 detectable CFU in the highest concentration plated on LB-Tet plates divided by the total CFU determined by plating on LB-Sm plates. HGT rates significantly higher than the limit of detection (LOD) were by evaluated by the Wilcoxon Signed Rank test against the hypothetical value of the LOD (*, *P* < 0.05, n=7-13). Significant differences between BEVs^VC1620/1::^*^tetR^* doses were analyzed via a Kruskal–Wallis test with uncorrected Dunn´s multiple comparison test (*, *P* < 0.05, n= 8).


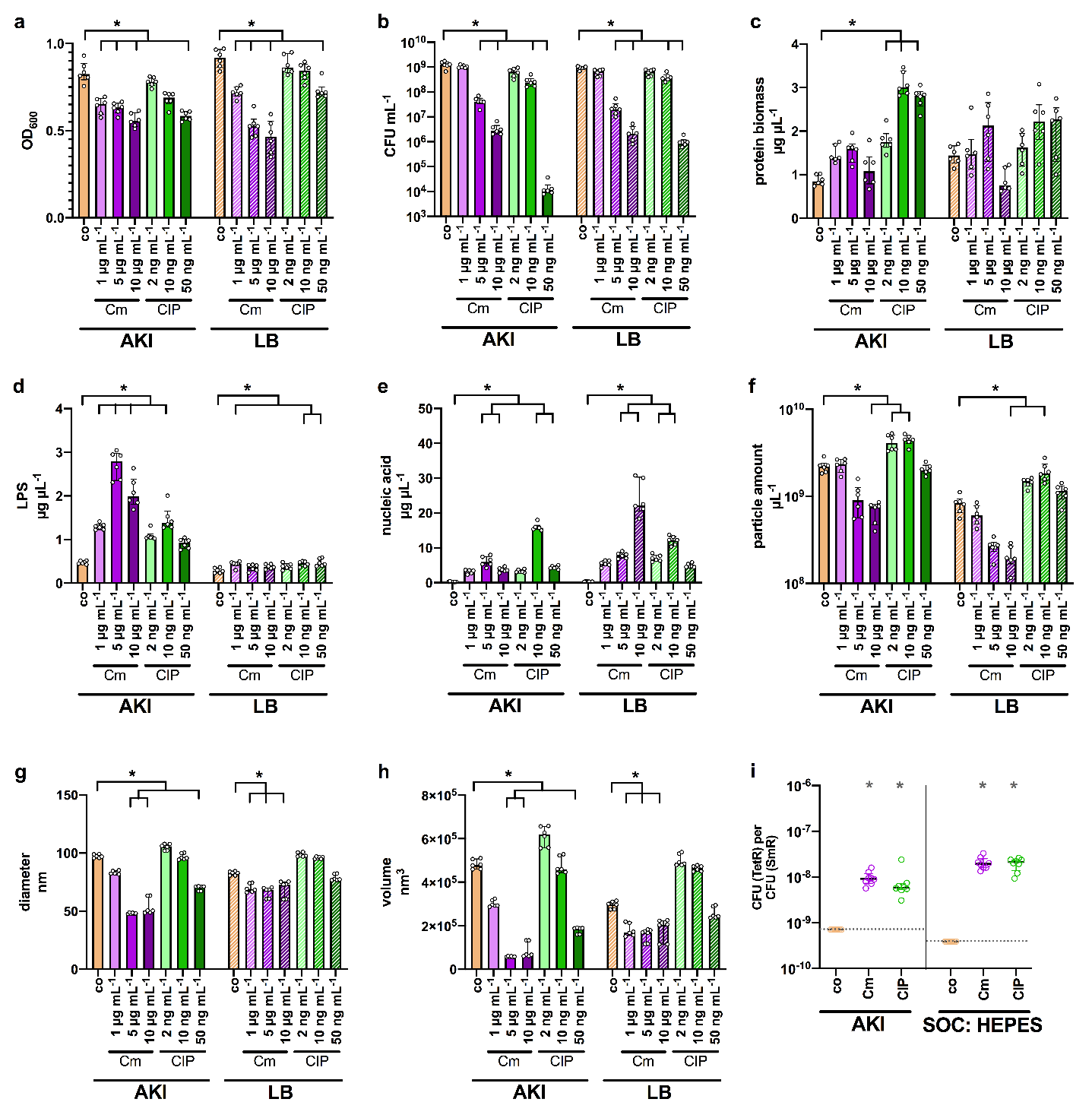


**Supplementary Figure S9: Isolation and characterization of BEVs derived from control and chloramphenicol and ciprofloxacin exposed *V. cholerae* cultures.**

**a and b,** Impact of chloramphenicol (Cm) and ciprofloxacin (CIP) on the growth of *V. cholerae* cultures used for BEV isolation. OD_600_ and CFU were assessed after growth in virulence (AKI) and non-virulence inducing conditions (LB) in absence (control, co) or presence of stressors (Cm or CIP) at the indicated concentrations.

**c,** Total protein biomass of the BEV preparations determined by Bradford.

**d,** LPS amount of the BEV preparations determined by purpald assay.

**e,** Nucleic acid amount of the BEV preparations determined by SYTO^TM^-9 staining using isolated chromosomal DNA of *V. cholerae* as standard.

**f,** Nanoparticle amount in the BEV preparations determined by nanoparticle tracking analysis (NTA).

**g,** Mean nanoparticle diameters of the BEV preparations determined by ZetaSizer Nano ZS90.

**h,** Volume of the BEVs calculated from the mean nanoparticle diameters determined via ZetaSizer Nano ZS90.

**a-h,** Data is presented as median ± interquartile range (IQR). For the AKI and LB data sets significant differences between the control and stress-induced samples were analyzed via a Kruskal–Wallis test with uncorrected Dunn´s multiple comparison (*, *P* < 0.05, n=6).

**i,** HGT rate of the *tetR*-cassette using BEVs derived from VC1620/1::*tetR* (BEVs^VC1620/1::^*^tetR^*) as donor. BEVs^VC1620/1::^*^tetR^* were isolated from AKI cultures in presence of Cm (5 µg mL^-1^) or CIP (10 ng mL^-1^) or without any stressor (control, co). *V. cholerae* WT grown in AKI or SOC: HEPES served as recipient and was incubated for 20 h at 30°C and 150 rpm with respective BEVs^VC1620/1::^*^tetR^* (2.5 x 10^11^ particles) before total CFU and transformants were determined by plating on LB-Sm and LB-Tet plates. Data is presented as median ± IQR. Assays yielding in no transformants on LB-Tet were set to limit of detection (LOD), which is indicated by a dotted line. The LOD was defined as 0.5 CFU detected in the highest concentration plated on LB-Tet plates divided by the total CFU determined by plating on LB-Sm plates. HGT rates significantly higher than the LOD were by evaluated by the Wilcoxon Signed Rank test against the hypothetical value of the LOD (*, *P* < 0.05, n=8).


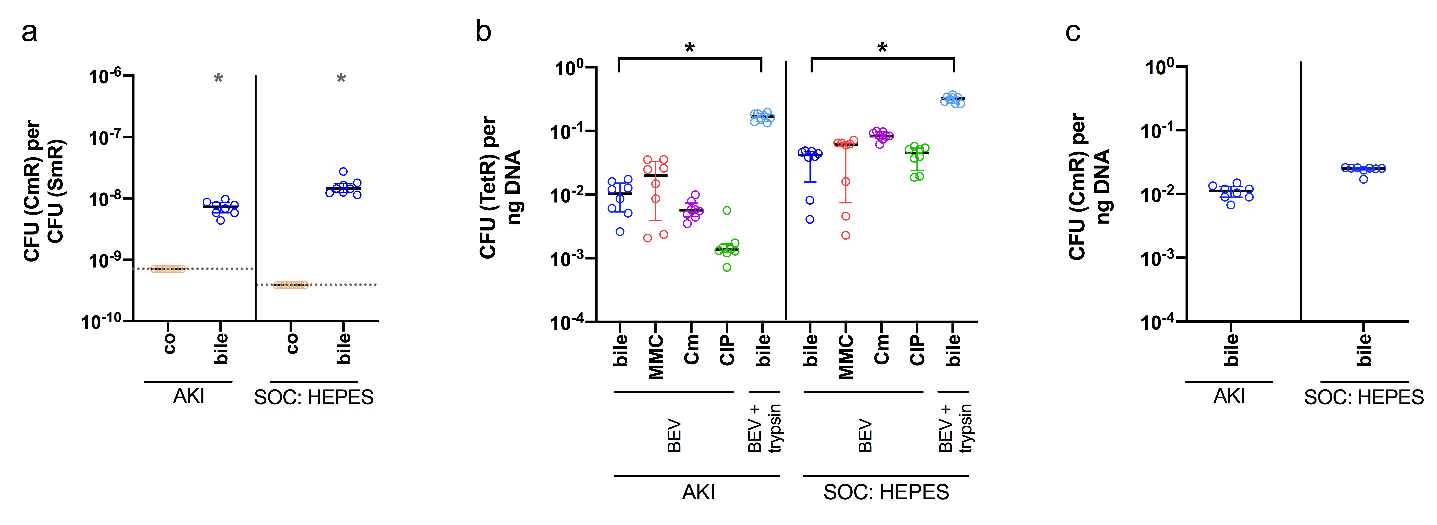


**Supplementary Figure S10: Additional BEV-mediated HGT data sets based on transfer of a CmR-cassette and presentation of DNA-normalized HGT rates.**

**a,** HGT rate of the *cmR*-cassette using BEVs derived from VC1620/1::*cmR* (BEVs^VC1620/1::^*^cmR^*) as donor. BEVs^VC1620/1::^*^cmR^* were isolated from AKI cultures in presence of bile (17.25 mM) or without any stressor (control, co). *V. cholerae* WT grown in AKI or SOC: HEPES served as recipient and was incubated for 20 h at 30°C and 150 rpm with respective BEVs^VC1620/1::^*^cmR^* (2.5 x 10^11^ particles) before total CFU and transformants were determined by plating on LB-Sm and LB-Cm plates. Data is presented as median ± IQR (n=8). Assays yielding in no transformants on LB-Tet were set to limit of detection (LOD), which is indicated by a dotted line. The LOD was defined as 0.5 CFU detected in the highest concentration plated on LB-Cm plates divided by the total CFU determined by plating on LB-Sm plates. HGT rates significantly higher than the LOD were by evaluated by the Wilcoxon Signed Rank test against the hypothetical value of the LOD (*, *P* < 0.05).

**b,** HGT rates normalized to the amount of DNA present in the BEVs for all assays of this study yielding in detectable transformants using stress-induced BEVs^VC1620/1::^*^tetR^* as donor. BEVs^VC1620/1::^*^tetR^* were isolated from AKI cultures in presence of bile (17.25 mM), MMC (60 ng mL^-1^), Cm (5 µg mL^-1^) or CIP (10 ng mL^-1^). In addition, trypsin digested BEVs^VC1620/1::^*^tetR^* derived from AKI cultures in presence of bile (17.25 mM) were used (see methods for detail). *V. cholerae* WT grown in AKI or SOC: HEPES served as recipient and was incubated for 20 h at 30°C and 150 rpm with respective BEVs^VC1620/1::^*^tetR^* (2.5 x 10^11^ particles) before transformants were determined by plating on LB-Tet plates and normalized against the BEV-associated DNA determined by SYTO^®^ 9 assays. Data is presented as median ± IQR (n=8). For the AKI and SOC:HEPES data sets significant differences between HGT rates using bile-induced BEVs^VC1620/1::^*^tetR^* before and after trypsin digest or HGT rates using different stress-induced BEVs were analyzed via Kruskal-Wallis test with uncorrected Dunn’s multiple comparisons (*, *P* < 0.05).

**c,** HGT rates normalized to the amount of DNA present in the BEVs for all assays of this study yielding in detectable transformants using bile-induced BEVs^VC1620/1::^*^cmR^* as donor. BEVs^VC1620/1::^*^cmR^* were isolated from AKI cultures in presence of bile (17.25 mM). *V. cholerae* WT grown in AKI or SOC: HEPES served as recipient and was incubated for 20 h at 30°C and 150 rpm with respective BEVs^VC1620/1::^*^cmR^* (2.5 x 10^11^ particles) before transformants were determined by plating on LB-Cm plates and normalized against the BEV-associated DNA determined by SYTO^®^ 9 assays. Data is presented as median ± IQR (n=8).

**References:**

1. Kolter R, Inuzuka M, Helinski DR. 1978. Trans-complementation-dependent replication of a low molecular weight origin fragment from plasmid R6K. Cell 15:1199-1208.

2. Roberts A, Pearson GD, Mekalanos JJ. Cholera vaccines strains derived from a 1991 Peruvian isolate of Vibrio cholerae and other El Tor strains, p. 43-47, p 43-47. *In* (ed),

3. Tamayo R, Schild S, Pratt JT, Camilli A. 2008. Role of cyclic Di-GMP during el tor biotype Vibrio cholerae infection: characterization of the in vivo-induced cyclic Di-GMP phosphodiesterase CdpA. Infect Immun 76:1617-27.

4. Baharoglu Z, Bikard D, Mazel D. 2010. Conjugative DNA transfer induces the bacterial SOS response and promotes antibiotic resistance development through integron activation. PLoS Genet 6:e1001165.

5. Donnenberg MS, Kaper JB. 1991. Construction of an *eae* deletion mutant of enteropathogenic *Escherichia coli* by using a positive-selection suicide vector. Infect Immun 59:4310-4317.

6. Bolivar F, Rodriguez RL, Greene PJ, Betlach MC, Heynecker HL, Boyer HW. 1977. Construction and characterization of a new cloning vehicle. II. A multipurpose cloning system. Gene 2:95-113.

7. Guzman L-M, Beblin D, Carson MJ, Beckwith J. 1995. Tight regulation, modulation, and high-level expression by vectors containing the arabinose pBAD promotor. J Bacteriol 177:4121-4130.

8. Kanehisa M, Goto S. 2000. KEGG: kyoto encyclopedia of genes and genomes. Nucleic Acids Res 28:27-30.
